# A TIAM1-TRIM28 complex mediates epigenetic silencing of protocadherins to promote migration of lung cancer cells

**DOI:** 10.1073/pnas.2300489120

**Published:** 2023-09-25

**Authors:** Lucy Ginn, Joe Maltas, Martin J. Baker, Anshuman Chaturvedi, Leah Wilson, Ryan Guilbert, Fabio M. R. Amaral, Lynsey Priest, Holly Mole, Fiona Blackhall, Zoi Diamantopoulou, Tim C. P. Somervaille, Adam Hurlstone, Angeliki Malliri

**Affiliations:** ^a^Cell Signalling Group, Cancer Research UK Manchester Institute, The University of Manchester, Manchester M20 4BX, United Kingdom; ^b^The Christie National Health Service Foundation Trust, Manchester M20 4BX, United Kingdom; ^c^Leukaemia Biology Laboratory, Cancer Research UK Manchester Institute, The University of Manchester, Manchester M20 4BX, United Kingdom; ^d^Division of Cancer Sciences, School of Medical Sciences, Faculty of Biology Medicine and Health, The University of Manchester, Manchester M13 9PT, United Kingdom; ^e^Division of Immunology, Infection and Respiratory Medicine, School of Biological Sciences, Faculty of Biology Medicine and Health, The University of Manchester, Manchester M13 9PT, United Kingdom

**Keywords:** TIAM1, RAC, migration, NSCLC, EMT

## Abstract

Lung cancer is the leading cause of cancer deaths. Its high mortality is associated with high metastatic potential. Here, we show that the RAC1-selective guanine nucleotide exchange factor T cell invasion and metastasis-inducing protein 1 (TIAM1) promotes cell migration and invasion in the most common subtype of lung cancer, non-small-cell lung cancer (NSCLC), through an unexpected nuclear function. We show that TIAM1 interacts with TRIM28, a master regulator of gene expression, in the nucleus of NSCLC cells. We reveal that a TIAM1-TRIM28 complex promotes epithelial-to-mesenchymal transition, a phenotypic switch implicated in cell migration and invasion. This occurs through H3K9me3-induced silencing of protocadherins and by decreasing E-cadherin expression, thereby antagonizing cell–cell adhesion. Consistently, TIAM1 or TRIM28 depletion suppresses the migration of NSCLC cells, while migration is restored by the simultaneous depletion of protocadherins. Importantly, high nuclear TIAM1 in clinical specimens is associated with advanced-stage lung adenocarcinoma, decreased patient survival, and inversely correlates with E-cadherin expression.

## Significance

Non-small-cell lung cancer (NSCLC) is mostly diagnosed at advanced stages of disease, characterized by epithelial-to-mesenchymal transition (EMT) of transformed lung cells. EMT is associated with enhanced migration and invasion of these cells but is also a mechanism of resistance to therapeutics and thus has multiple effects on disease progression and treatment outcomes. At present, we have an incomplete understanding of how this dramatic reprogramming of cell phenotype is achieved. In this study, we demonstrate that TIAM1, an activator of the small GTPase RAC1, promotes EMT and migration of NSCLC cells when localized in the nucleus and identify the molecular mechanism behind this. Moreover, we find that high nuclear TIAM1 is associated with advanced-stage lung adenocarcinoma and decreased survival.

Non-small-cell lung cancer (NSCLC) accounts for approximately 85% of lung cancer cases (1). Lung adenocarcinoma (LUAD), which arises from the alveolar epithelium, is the most common histological subtype of NSCLC (2). NSCLC is a relatively chemo-resistant disease which is typically diagnosed at advanced stages when distal and secondary metastases are common and is associated with high mortality rates (3, 4). A key process promoting the metastatic potential of carcinoma is epithelial-to-mesenchymal transition (EMT), which is implicated in the acquisition of cell motility and invasiveness, as well as therapeutic resistance (5, 6). Recently, therapeutics against the small GTPase KRAS, the most commonly mutated oncoprotein in LUAD, have been developed, but resistance is common (7–10). EMT was shown to be a mechanism of both acquired and intrinsic resistance to inhibitors of the most prevalent KRAS mutation in NSCLC cells, KRAS^G12C^ (11). However, despite its importance in carcinoma progression and resistance, the precise mechanisms responsible for EMT in NSCLC are not fully known. Therefore, it is important to gain a deeper understanding of pathways which regulate EMT in NSCLC to direct future therapies and reduce disease burden.

The RAC1 signaling pathway regulates key cellular processes, including cytoskeletal dynamics, polarity, adhesion, and extracellular matrix remodeling (12, 13) which are all important aspects of cell migration, invasion, and EMT. RAC1 signaling is frequently deregulated in cancer through mutations or overexpression of RAC1 guanine nucleotide exchange factors (GEFs) or RAC1 itself (14, 15). Alternatively, this can also occur because of the activation of upstream regulators such as KRAS (12). Interestingly, in NSCLC, RAC1 is required for oncogenic KRAS-induced lung cancer in mice (16). Moreover, RAC1 signaling has been shown to promote EMT in lung cancer (17), as well as resistance to chemotherapy and targeted therapies in several cancers (18). Although directly targeting RAC1 with therapeutics is actively being pursued, the diverse physiological roles of RAC1 (12) make such therapeutics likely to be toxic in patients. Furthermore, activation of RAC1 can have contrasting effects on cell migration (19, 20). Therefore, selective intervention in targeting RAC1 may be needed to circumvent the potential undesirable effects of its direct inhibition (21). Interestingly, we have previously shown that RAC1 GEFs not only activate RAC1 but also work as scaffold proteins to orchestrate downstream signaling, therefore providing selectivity in RAC1 signaling (20). Consequently, targeting the activation of RAC1 by particular GEFs should be a better approach than targeting RAC1 itself.

The RAC1 GEF TIAM1 is a candidate therapeutic target as TIAM1 is a RAS effector required for oncogenic RAS-induced skin cancer (22, 23). Moreover, TIAM1 knockout mice are viable with no apparent phenotype, indicating that TIAM1-RAC1 inhibitors in patients would likely be tolerated (22). However, TIAM1 also exhibits context-dependent roles in cancer. For example, in contrast to skin cancer, where TIAM1 is required for RAS-induced tumor initiation and growth but antagonizes malignant conversion (22), in pancreatic cancer, TIAM1-RAC1 signaling promotes both proliferation and invasion (24). Furthermore, TIAM1-dependent cell behaviors are influenced by the distinct subcellular localizations of TIAM1 (25, 26). For example, the ability of TIAM1 to suppress cell migration and invasion has been attributed to its localization at cell–cell adhesions and its role in establishing and maintaining adherens junctions (AJs) (20, 27, 28). In keeping, we have previously shown that Src-induced cell scattering requires depletion of TIAM1 specifically from AJs (29). Moreover, we have more recently shown that TIAM1 present in the nucleus of colorectal cancer (CRC) cells also inhibits migration. This is because nuclear TIAM1 suppresses the interaction of YAP/TAZ transcriptional coactivators with TEA domain (TEAD) transcription factors, inhibiting the expression of YAP/TAZ target genes implicated in cell migration (30). Consistent with these in vitro findings, high nuclear TIAM1 in clinical specimens was associated with increased CRC patient survival (30). Therefore, it is important to understand the role of TIAM1 and its interactors in different cancer contexts to exploit its therapeutic potential.

There is limited evidence implicating TIAM1 in NSCLC. In an EGFR mutant lung cancer setting, TIAM1 was shown to be required for proliferation and tumor growth (31). Here, we identified that in contrast to other cancers of epithelial origin, TIAM1 is required for the migration and invasion of NSCLC cells. Importantly, we show that TIAM1 controls the migration of NSCLC cells from the nucleus and identified the molecular mechanism behind this regulation, which involves TIAM1 being part of the TRIM28-SETDB1 transcriptional repressor complex suppressing the expression of cell–cell adhesion proteins and consequently promoting EMT and migration.

## Results

### Nuclear TIAM1 Increases in Late-Stage NSCLC Tumors and Is Required for Optimal Cell Migration and Invasion.

**Fig. 1.**
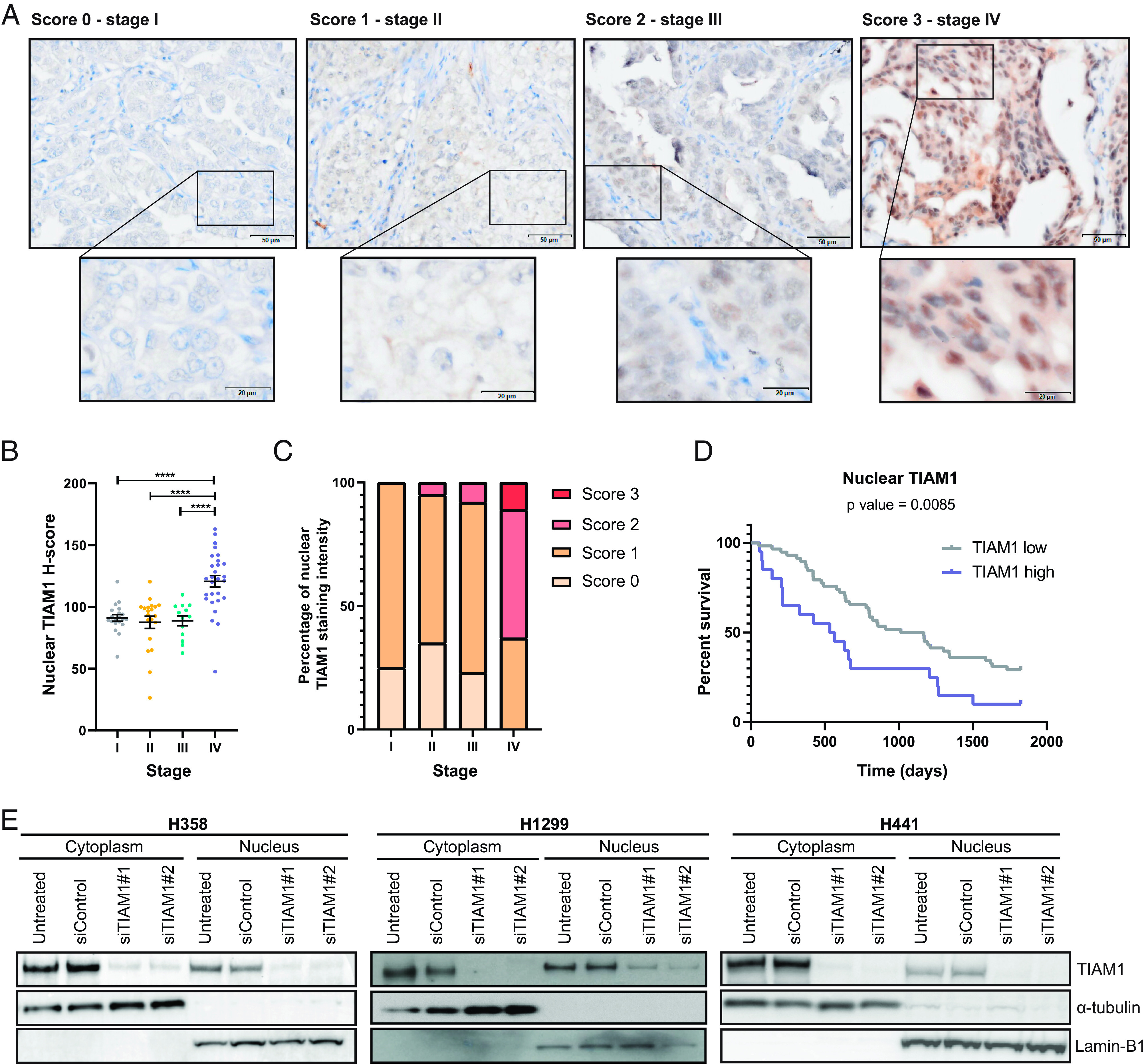
Abundance of nuclear TIAM1 correlates with NSCLC progression. (*A*) Representative examples of strong (score 3), moderate (score 2), weak (score 1), and negative (score 0) immunohistochemical staining of TIAM1 expression in a panel of LUAD tumors from stage I-IV patients. (Scale bars, 50 μm and 20 μm.) (*B* and *C*) Quantification of nuclear TIAM1 staining intensity from samples as in (*A*), using HALO AI image analysis software to calculate TIAM1 H-score per patient (*B*) or histopathologist analysis of the percentage of overall TIAM1 staining score (0 to 3) per patient (*C*) per stage. Stage I, n = 20; stage II, n = 20; stage III, n = 13, and stage IV, n = 28 patients. (*D*) Kaplan–Meier plot comparing the 5-y survival of patients with the highest quartile of nuclear TIAM1 expression vs. the other three quartiles (the *P* value shown is for the Mantel–Cox test). (*E*) Western blot analysis of TIAM1 protein levels in the cytoplasmic and nuclear fractions of H358, H1299, and H441 cells that were untreated or transfected with TIAM1 or control siRNAs, as indicated. Lamin B1 and α-tubulin were used as nuclear and cytoplasmic loading controls, respectively. ****P < 0.0001 (ANOVA).

There is some evidence that TIAM1 is overexpressed in lung adenocarcinoma (LUAD) tumors from patients (31, 32); however, the subcellular localization of TIAM1 and its potential correlation with disease progression have not been evaluated. To address this, we probed a panel of LUAD tumors from stage I-IV patients with a prevalidated TIAM1 antibody (30, 33). We detected TIAM1 not only in the cytoplasm but also in cell nuclei (Fig. 1*A*). Interestingly, the staining intensity for nuclear TIAM1 is increased at advanced disease when analyzed both by AI and by a histopathologist (Fig. 1 *B* and *C* and *SI Appendix*, Fig. S1*A*). In contrast, cytoplasmic TIAM1 did not show a strong increase with stage (*SI Appendix*, Fig. S1*B*). Thus, nuclear TIAM1 expression is positively associated with LUAD progression. To determine whether TIAM1 expression is associated with overall survival of patients, we separated LUAD patients based on the expression of nuclear or cytoplasmic TIAM1. We found that patients with high nuclear TIAM1 have significantly worse survival than patients with low nuclear TIAM1 staining, whereas cytoplasmic TIAM1 did not correlate with survival (Fig. 1*D* and *SI Appendix*, Fig. S1*C*). Therefore, nuclear TIAM1 could serve as a prognostic factor for LUAD patients.

To validate the nuclear localization of TIAM1 in NSCLC, we analyzed its expression and localization in three NSCLC cell lines: H358, H1299, and H441. Consistent with the clinical findings, TIAM1 was detected in the cytoplasm and the nucleus of these cell lines by both biochemical fractionation and immunofluorescence (Fig. 1*E* and *SI Appendix*, Fig. S1*D*). Furthermore, TIAM1 depletion using two different small interfering RNAs (siRNAs) dramatically reduced both cytoplasmic and nuclear TIAM1 (Fig. 1*E* and *SI Appendix*, Fig. S1*D*), validating the specificity of the staining. As nuclear TIAM1 expression was increased at advanced stages of the disease and high nuclear TIAM1 expression correlated with worse survival, we focused on the role of nuclear TIAM1 in NSCLC.

**Fig. 2.**
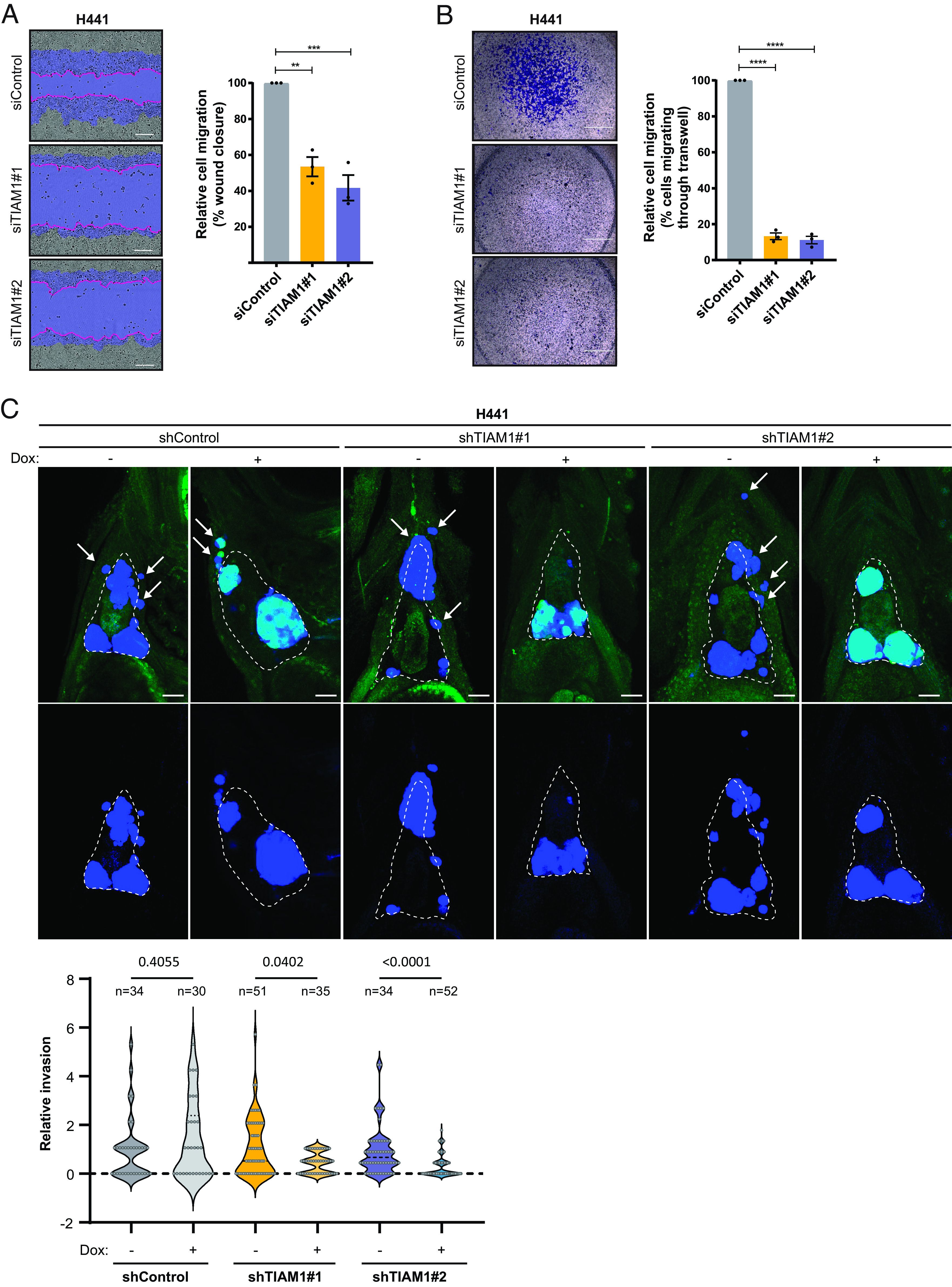
TIAM1 depletion reduces NSCLC cell migration and invasion in vitro and in vivo. (*A*) Representative images of H441 cells transfected with TIAM1 or control siRNAs migrating over a cell-free zone (wound). The initial wound area (time = 0 h) is shaded blue, and the final wound area (time = 12 h) is demarcated by the pink line. (Scale bar, 200 μm.) The graph shows percentage wound closure relative to initial wound size, where control cell migration is set to 100%. (*B*) Representative images of H441 cells transfected with TIAM1 or control siRNAs migrating through Transwell inserts. (Scale bar, 1,000 μm.) The graph shows the average percentage of migrating cells where control cell migration is set to 100%. (*C*) Representative max intensity projections of H441 xenografts in *nacre^−/−^* zebrafish embryos at 3 d after injection, treated without (−) or with (+) doxycycline (Dox) to induce shRNA and Green Fluorescent Protein (GFP) expression. Top panels show Cell Trace Violet staining (blue) of implanted H441 cells overlaid on the tissue autofluorescence and GFP signal (green). Bottom panels show Cell Trace Violet signal only. The outline of the pericardial cavity, as determined by tissue autofluorescence, is indicated in each image and representative invaded cells indicated by arrows. (Scale bar, 50 μm.) The graph shows the fold-change in the number of cells that invaded per fish for doxycycline-treated versus untreated for each cell line. Statistical significance was tested using a one-way ANOVA with the Kruskal–Wallis multiple comparison test, controlling the FDR with the Benjamini, Krieger, and Yekutieli method. Data are presented as mean ± SEM of three independent experiments. **P < 0.01, ***P < 0.001, and ****P < 0.0001 (ANOVA).

Late-stage tumors are characterized by increased migration and invasion of tumor cells. Given the increased expression of nuclear TIAM1 at late stages of LUAD, we investigated the effect of TIAM1 on the migration of NSCLC cells. Using two different siRNAs and two different migration assays [Boyden Chamber assays testing migration in response to transforming growth factor β (TGFβ) and scratch wound assays], we found that TIAM1 depletion significantly reduced the migration of H441 and H358 cells (Fig. 2 *A* and *B* and *SI Appendix*, Fig. S2 *A* and *B*), without affecting their proliferation (*SI Appendix*, Fig. S2*C*). Furthermore, TIAM1 depletion in H441 cells reduced cell migration and invasion in response to hepatocyte growth factor (HGF) (*SI Appendix*, Fig. S2*D*). To assess the role of TIAM1 on migration and invasion in vivo, we used a zebrafish embryo xenograft model, using doxycycline-inducible TIAM1 knockdown H441 cells (*SI Appendix*, Fig. S2*E*). Cells were injected into the pericardial cavity of zebrafish embryos 2 d after fertilization, and dissemination outside the cavity was quantified 72 h later (*SI Appendix*, Fig. S2*F*). Significantly fewer TIAM1-depleted H441 cells invaded compared with control cells (Fig. 2*C*). Taken together, these results demonstrate that TIAM1 suppresses the migration and invasion of NSCLC cells both in vitro and in vivo.

**Fig. 3.**
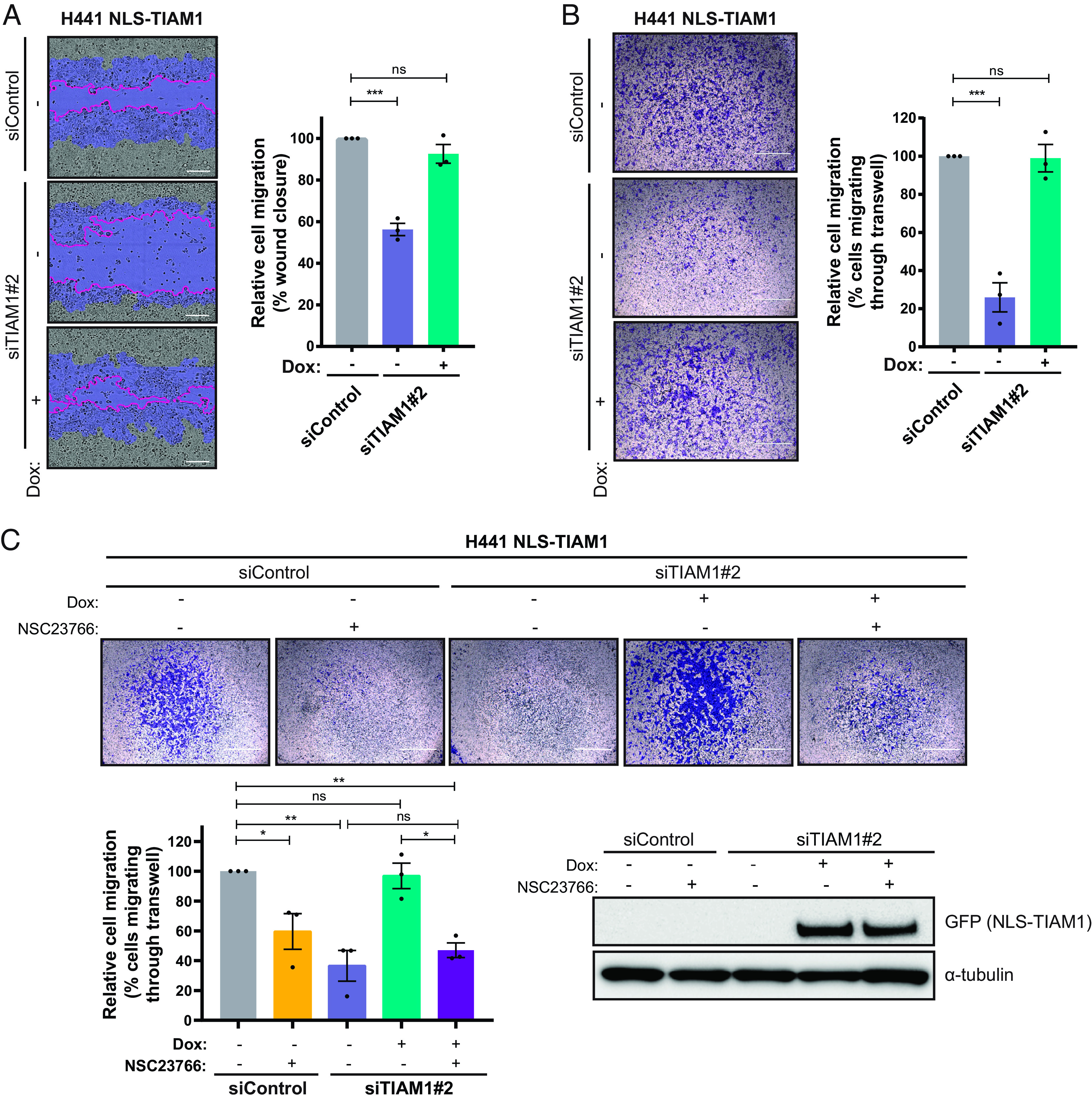
Nuclear TIAM1 promotes NSCLC cell migration dependent on RAC1. (*A*) Representative images of H441 cells migrating through a wound shaded as in 2A, transfected with TIAM1 or control siRNAs and inducibly expressing NLS-TIAM1 (resistant to siTIAM1#2), following the addition of doxycycline. (Scale bar, 200 μm.) The graph shows percentage wound closure relative to initial wound size. (*B*) Representative images of H441 cells migrating through Transwell inserts, transfected with TIAM1 or control siRNAs and inducibly expressing NLS-TIAM1 following the addition of doxycycline. (Scale bar, 1,000 μm.) The graph shows average percentage of migrating cells where control cell migration is set to 100%. (*C*) Representative images of H441 cells migrating through Transwell inserts, transfected with TIAM1 or control siRNAs and inducibly expressing NLS-TIAM1 following the addition of doxycycline. Cells were treated with 20 μM NSC23766 (+) or DMSO control (−). (Scale bar, 1,000 μm.) The graph shows the average percentage of migrating cells relative to siControl cells, normalized to cell viability. Data are presented as mean ± SEM of three independent experiments. ns = nonsignificant, *P < 0.05, **P < 0.01, and ***P < 0.001 (ANOVA).

To investigate specifically the role of nuclear TIAM1 on migration, rescue experiments were performed using TIAM1 forced to the nucleus (NLS-TIAM1) by the well-characterized nuclear localization signal (NLS) of SV40 large T antigen (30). We verified that the NLS-TIAM1 construct (resistant to siTIAM1#2) was exclusively nuclear by confocal microscopy in H441 and H358 cells (*SI Appendix*, Fig. S3*A*). Interestingly, NLS-TIAM1 fully rescued the reduced migration of TIAM1-depleted NSCLC cells (Fig. 3 *A* and *B* and *SI Appendix*, Fig. S3 *A–C*), indicating that nuclear TIAM1 promotes the migration of these cells. Moreover, cytoplasmic localized TIAM1 (NES-TIAM1, resistant to siTIAM1#2) was no longer able to rescue the decrease in migration observed with TIAM1 knockdown (*SI Appendix*, Fig. S3 *D–F*), further supporting a role for nuclear TIAM1 in promoting NSCLC migration.

TIAM1 is known to mediate its effects primarily through the activation of RAC1 (34–37). To investigate the importance of RAC1 in nuclear TIAM1-mediated migration, we treated cells with the RAC1 inhibitor NSC23766, which inhibits the activation of RAC1 by certain GEFs, including TIAM1 (38). As expected, the RAC1 inhibitor led to a reduction in H441 migration in a dose-dependent manner (*SI Appendix*, Fig. S3*G*). Furthermore, in cells treated with the RAC1 inhibitor, expression of nuclear-localized TIAM1 was no longer able to reverse the decrease in migration observed with TIAM1 knockdown (Fig. 3*C*), suggesting that nuclear TIAM1 requires RAC1 activity to promote NSCLC cell migration. Importantly, using a previously described “GEF-dead” TIAM1 mutant (GD-TIAM1) (20, 37, 39, 40), we were able to assess whether the GEF activity of TIAM1 was required to promote migration. Both NLS- and NLS-GD-TIAM1 proteins were localized to the nucleus following addition of doxycycline (*SI Appendix*, Fig. S3*H*), and again, TIAM1 depletion reduced migration in both NLS- and NLS-GD-TIAM1 control cells (without addition of doxycycline) (*SI Appendix*, Fig. S3 *I* and *J*). However, only cells expressing nuclear localized TIAM1 with a functional GEF domain were able to significantly increase cell migration following TIAM1 depletion (*SI Appendix*, Fig. S3 *I* and *J*). Therefore, activation of RAC1 by TIAM1 in the nucleus is required for optimal cell migration of NSCLC cells.

### TIAM1 Interacts with TRIM28 and SETDB1 to Regulate Methylation of Histone H3 at Lysine 9.

To address the mechanism by which nuclear TIAM1 promotes migration of NSCLC cells, we performed two proteomic screens to identify interactors of nuclear TIAM1: immunoprecipitation of TIAM1 from the nucleus of NSCLC cells and a BioID (proximity-dependent biotin identification) screen, both followed by mass spectrometry (*SI Appendix*, Fig. S4*A*). For the BioID screen, NLS-TIAM1 was tagged with the mutant biotin ligase (BirA*) (NLS-TIAM1-BirA*). This, as well as negative control constructs (BirA*, NLS-BirA*, TIAM1, and NLS-TIAM1), were inducibly expressed at physiological levels in H1299 NSCLC cells (*SI Appendix*, Fig. S4 *B* and *C*). Next, cells were supplemented with biotin, and the activity of BirA* was confirmed by analyzing the levels of protein biotinylation by western blot (*SI Appendix*, Fig. S4*D*). The NLS tag induced a predominantly nuclear localization of TIAM1-BirA* (*SI Appendix*, Fig. S4*E*). The BirA* only control was also mainly localized to the nucleus (*SI Appendix*, Fig. S4*E*). Moreover, TIAM1-BirA*, which localized to both the cytoplasm and the nucleus, was able to biotinylate proteins within both subcellular compartments following the addition of biotin, while NLS-TIAM1-BirA* biotinylated predominantly nuclear proteins (*SI Appendix*, Fig. S4*F*).

**Fig. 4.**
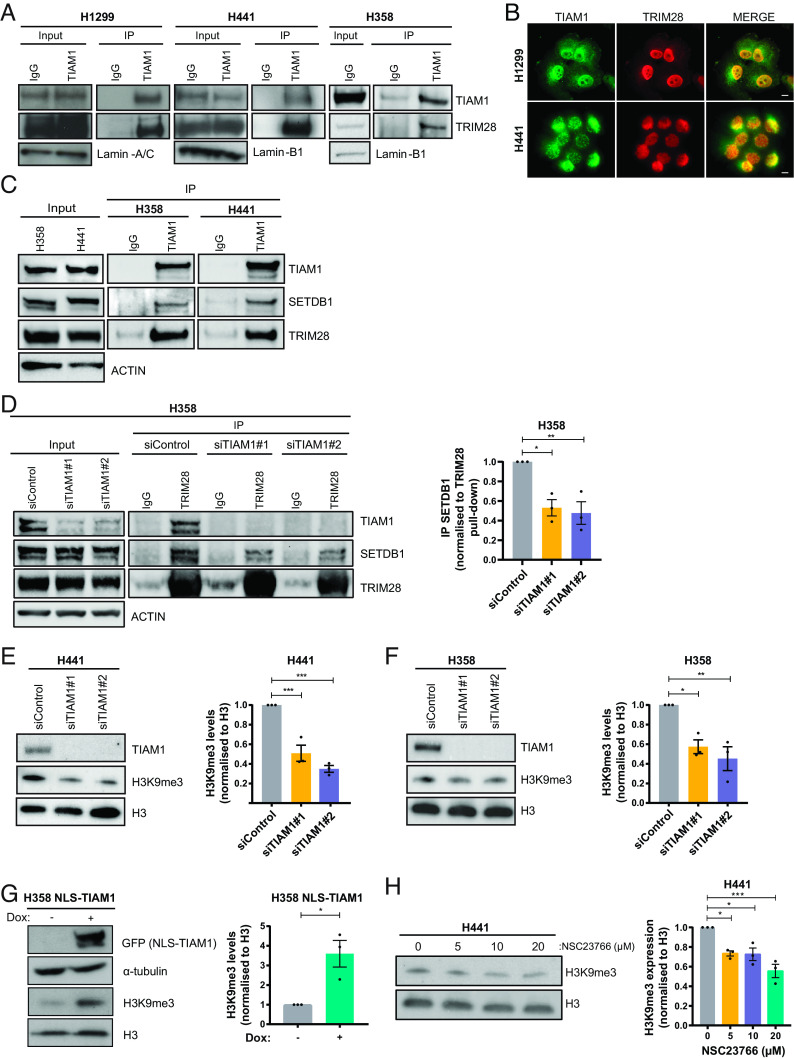
TIAM1 interacts with TRIM28 and SETDB1 to regulate H3K9 trimethylation. (*A*) Western blot analysis of endogenous TRIM28 co-immunoprecipitating with endogenous TIAM1 from the nuclear fraction of H1299, H441, and H358 cells. (*B*) Immunofluorescence images of H1299 and H441 cells stained for DNA with DAPI (blue), TIAM1 (green), and TRIM28 (red) with a merged overlay. (Scale bar, 20 μm.) (*C*) Western blot analysis of endogenous SETDB1 and TRIM28 co-immunoprecipitating with endogenous TIAM1. (*D*) Western blot analysis of endogenous SETDB1 co-immunoprecipitating with endogenous TRIM28, in cells transfected with TIAM1 or control siRNAs. The graph quantifies immunoprecipitated SETDB1 levels relative to immunoprecipitated TRIM28. (*E* and *F*) Western blot analysis of TIAM1 and H3K9me3 levels in H441 (*E*) and H358 (*F*) cells transfected with TIAM1 or control siRNAs. Graphs show H3K9me3 levels normalized to Histone H3. (*G*) Western blot analysis of NLS-TIAM1-GFP and H3K9me3 levels in uninduced control (−) H358 cells or cells with doxycycline-induced (+) expression of NLS-TIAM1. The graph shows H3K9me3 levels normalized to Histone H3. (*H*) Western blot analysis of H3K9me3 levels in H441 cells treated with an increasing dose of NSC23766. The graph shows H3K9me3 levels normalized to Histone H3. Data are presented as mean ± SEM of three independent experiments. *P < 0.05, **P < 0.01, and ***P < 0.001 (ANOVA).

One TIAM1 interactor identified in both screens was the transcriptional corepressor tripartite motif containing 28 (TRIM28) (also known as KAP1 and TIF1β) (*SI Appendix*, Fig. S4*G*). The interaction between endogenous TIAM1 and endogenous TRIM28 was confirmed by immunoprecipitation of TIAM1 from nuclear fractions of H1299, H441, and H358 cells (Fig. 4*A*). Furthermore, immunofluorescence of TIAM1 and TRIM28 revealed localization of the two proteins in the nucleus (Fig. 4*B*). TRIM28 is known to function in complex with the histone methyltransferase SET Domain Bifurcated Histone Lysine Methyltransferase 1 (SETDB1) to regulate gene expression via repressive histone marks (41). As expected, therefore, immunoprecipitation of endogenous TIAM1 pulled down SETDB1, as well as TRIM28, in H358 and H441 cells from both whole cell extracts and nuclear extracts (Fig. 4*C* and *SI Appendix*, Fig. S5*A*). To investigate whether TIAM1 has a role in the formation of this repressor complex, we performed further immunoprecipitation experiments in TIAM1- or TRIM28-depleted cells. Interestingly, TIAM1 depletion led to a reduction in the amount of SETDB1 that co-immunoprecipitated with TRIM28 (Fig. 4*D*), whereas TRIM28 depletion did not alter the levels of SETDB1 that co-immunoprecipitated with TIAM1 (*SI Appendix*, Fig. S5*B*). Furthermore, using previously engineered TIAM1 deletion mutants (40), both the C and N terminus of TIAM1 were shown to interact with TRIM28 (*SI Appendix*, Fig. S5 *C* and *D*). These data suggest that TIAM1 may therefore be acting as a scaffold for TRIM28 and SETDB1 interaction.

Due to the roles of the TRIM28-SETDB1 repressor complex in regulating mainly the levels of histone 3 trimethylation on lysine 9 (H3K9me3) (42, 43), we next investigated the interplay of TIAM1 and H3K9me3 levels. Interestingly, TIAM1 depletion using two independent siRNAs led to a significant reduction in global H3K9me3 levels in H441 and H358 cells (Fig. 4 *E* and *F*). Furthermore, overexpression of NLS-TIAM1 in H358 cells (*SI Appendix*, Fig. S5*E*) led to a significant increase in the global levels of H3K9me3 (Fig. 4*G*). The striking correlation between nuclear TIAM1 and H3K9me3 levels prompted us to investigate whether TIAM1 was present in the chromatin. Indeed, subcellular fractionation identified chromatin-bound TIAM1 in H441 cells (*SI Appendix*, Fig. S5*F*). Interestingly, RAC1 inhibition also decreased H3K9me3 levels in H441 cells comparable to that seen after TIAM1 depletion (Fig. 4*H*), suggesting a role for RAC1 in regulating H3K9me3 levels. Taken together, these results show that TIAM1 is part of the repressive complex consisting of TRIM28 and SETDB1 and regulates repressive histone marks.

### TIAM1 Regulates the Expression of Cell–Cell Adhesion Genes.

**Fig. 5.**
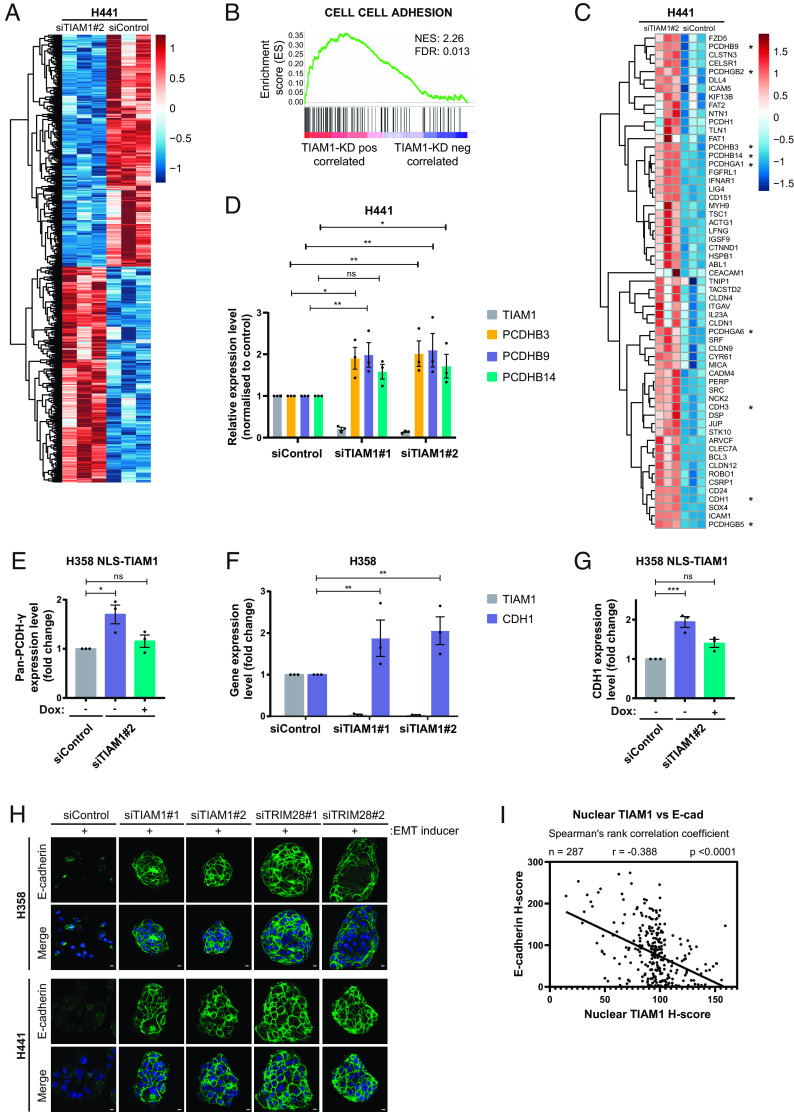
TIAM1 regulates the expression of cell–cell adhesion genes. (*A*) Heatmap depiction of differentially expressed genes between H441 cells transfected with TIAM1 or control siRNAs and treated with EMT inducer. (*B*) GSEA showing that cell–cell adhesion genes are positively correlated with TIAM1 knockdown in H441 cells. FDR and normalized enrichment score (NES) are shown. (*C*) Heatmap depiction of the core enrichment genes that contribute to the cell–cell adhesion signature shown in *B*. Classical cadherin members and clustered PCDHs are indicated with an asterisk. (*D*) qRT-PCR analysis of PCDHB3, PCDHB9, and PCDHB14 expression levels in H441 cells transfected with TIAM1 or control siRNAs. The graph shows expression levels normalized to control. (*E*) qRT-PCR analysis of pan-PCDH-γ expression level in H358 cells transfected with TIAM1 or control siRNAs and treated with (+) or without (−) doxycycline to induce NLS-TIAM1 expression. The graph shows expression levels normalized to control. (*F*) qRT-PCR analysis of CDH1 and TIAM1 expression levels in H358 cells transfected with TIAM1 or control siRNAs and treated with EMT inducer. The graph shows expression levels normalized to control. (*G*) qRT-PCR analysis of CDH1 expression in H358 cells transfected with TIAM1 or control siRNAs, treated with EMT inducer and with (+) or without (−) doxycycline to induce NLS-TIAM1 expression. The graph shows expression levels normalized to control. (*H*) Immunofluorescence images of H358 and H441 cells stained with DAPI (blue) and for E-cadherin (green) with a merged overlay, treated with EMT inducer and transfected with TIAM1, TRIM28 or control siRNAs. (Scale bar, 10 μm.) (*I*) Correlation analysis of nuclear TIAM1 H-score vs. E-cadherin H-score in 287 human LUAD IHC samples. Spearman's rank correlation coefficient was used to generate r and *P* values; linear regression was used to generate the trend line. Data are presented as mean ± SEM of three independent experiments. ns = nonsignificant, *P < 0.05, **P < 0.01, and ***P < 0.001 (ANOVA).

The nuclear localization of TIAM1, its interaction with TRIM28 and SETDB1, and the regulation of histone methylation suggested a possible role for TIAM1 in regulating gene expression. Moreover, it has previously been shown that TRIM28 promotes TGF-β-induced EMT of NSCLC cells by increasing H3K9me3 levels at the E-cadherin promoter and consequently silencing E-cadherin expression (42). We therefore investigated the requirement for TIAM1 in regulating gene expression of NSCLC cells treated with EMT-inducing factors. For this, H441 cells were treated with an EMT inducer cocktail containing TGF-β to disrupt cell–cell adhesion (*SI Appendix*, Fig. S6*A*) and were also transfected with either TIAM1-targeting or control siRNAs (*SI Appendix*, Fig. S6*B*). RNA sequencing (RNA-seq) was then performed to identify TIAM1 differentially expressed genes. A total of 4,543 genes were found to be differentially expressed [False discovery rate (FDR) < 0.05] between the two populations (Fig. 5*A* and Dataset S1).

To define molecular pathways associated with TIAM1-regulated genes, we performed gene set enrichment analysis (GSEA) using gene sets in the Molecular Signature Database (MSigDB) (44). GSEA revealed significant enrichment (normalized enrichment score = 2.26) among TIAM1-regulated genes for genes associated with cell–cell adhesion (FDR <0.05) (Fig. 5*B*). Indeed, several cadherin family members belonging to the classical cadherin (CDH1 and CDH3) and clustered protocadherin (PCDH) (PCDHB3, PCDHB9, PCDHB14, PCDHGA1, PCDHGA6, PCDHGB2, and PCDHGB5) subfamilies were found to be up-regulated in the RNA-seq dataset upon knockdown of TIAM1 (Fig. 5*C*). The upregulation of PCDHB3, PCDHB9, and PCDHB14 was validated by qRT-PCR in H441 cells using two independent siRNA sequences to target TIAM1 (Fig. 5*D*). To investigate specifically the role of nuclear TIAM1 on protocadherin expression, NLS-TIAM1 (resistant to siTIAM1#2) was expressed at levels comparable with endogenous nuclear TIAM1 in H358 cells after TIAM1 depletion (*SI Appendix*, Fig. S6*C*). Using pan-PCDH-γ primers for the constant region of the PCDH-γ genes, we found that NLS-TIAM1 restored the expression of pan-PCDH-γ in TIAM1-depleted cells to levels observed in control cells (Fig. 5*E*).

Similarly, we validated the upregulation of CDH1 following TIAM1 depletion in H358 cells treated with EMT inducer (Fig. 5*F*), an effect that could be rescued by the expression of NLS-TIAM1 (Fig. 5*G*). As TRIM28 is known to regulate the protein levels of E-cadherin through suppression of CDH1 gene transcription (42), we next investigated whether TIAM1-mediated regulation of CDH1 expression translated into changes in protein level. Interestingly, we found a reduction in E-cadherin protein levels in H441 cells following their treatment with EMT inducer, which was rescued by depletion of TIAM1 or TRIM28 (*SI Appendix*, Fig. S6*D*). Immunofluorescence for E-cadherin was also performed following TIAM1 and TRIM28 depletion in H358 and H441 cells in the presence of an EMT inducer cocktail. Interestingly, E-cadherin was visibly localized at cell–cell adhesions of cells depleted for TIAM1 or TRIM28 in contrast to control cells (Fig. 5*H*). Correspondingly, we observed a compact clustered epithelial cell morphology following depletion of either TIAM1 or TRIM28, suggesting that TIAM1 and TRIM28 are required for the induction and maintenance of EMT (*SI Appendix*, Fig. S6*E*). In addition, we observed that the combined depletion of TIAM1 and TRIM28 did not have an additive effect on E-cadherin protein levels in H441 cells, indicating that these proteins likely act in the same pathway to regulate E-cadherin (*SI Appendix*, Fig. S6*F*). Importantly, probing the LUAD clinical samples described in Fig. 1 for expression of E-cadherin revealed that nuclear TIAM1 and E-cadherin are inversely correlated in LUAD samples (Fig. 5*I* and *SI Appendix*, Fig. S6 *G* and *H*). Therefore, we conclude that TIAM1 regulates the expression of E-cadherin and PCDHs to disrupt cell–cell adhesion and promote a migratory phenotype in NSCLC cells.

### TIAM1 Binds the Protocadherin Genomic Cluster and Promotes NSCLC Cell Migration by Suppressing the Expression of Protocadherins.

**Fig. 6.**
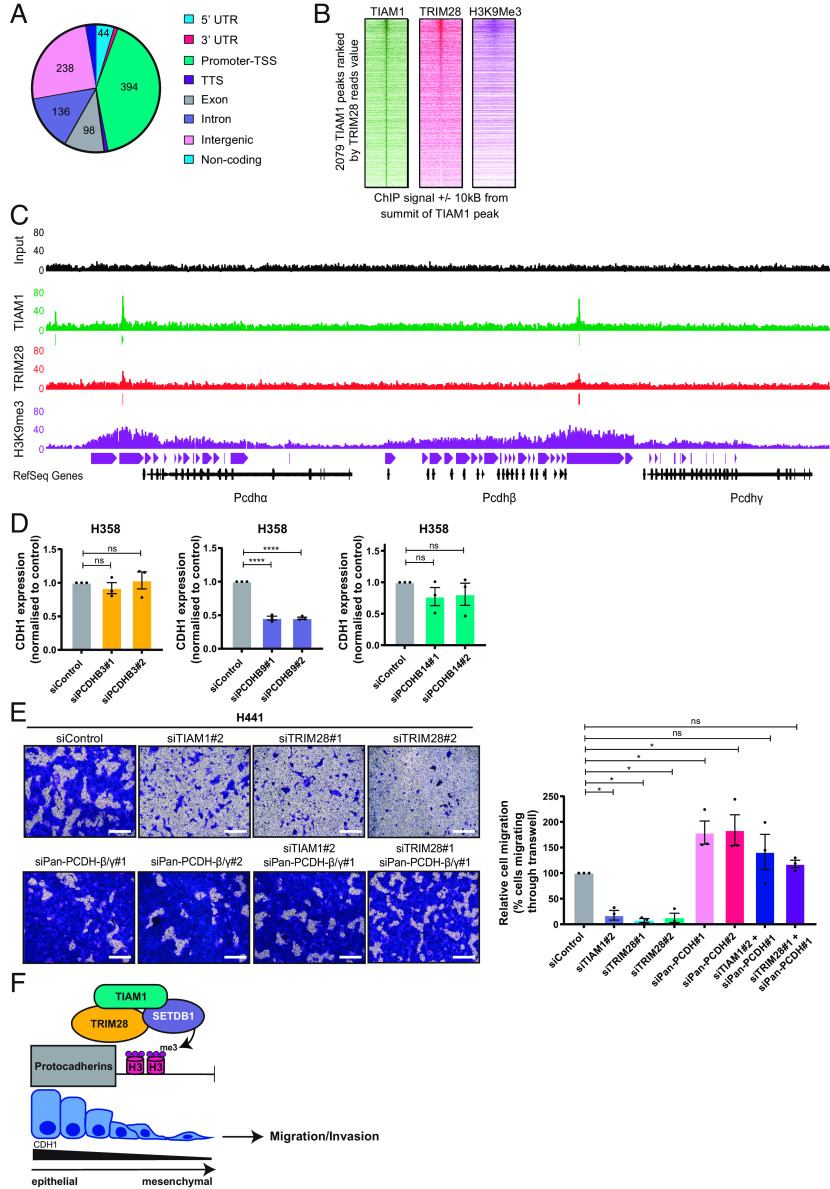
TIAM1 directly regulates protocadherin expression and thereby cell migration. (*A*) Pie chart showing the number of TIAM1 binding peaks at different genomic regions identified by TIAM1 ChIP-seq in H441 cells. (*B*) Heatmap depiction of TIAM1 peaks in the chromatin ranked by TRIM28 and deposition of H3K9me3 marks. (*C*) Read density tracks of TIAM1, TRIM28, and H3K9me3 ChIP-seq at the clustered protocadherin loci of H441 cells, with input sample shown for reference. (*D*) qRT-PCR analysis of CDH1 expression in H358 cells transfected with PCDHB3, PCDHB9, PCDHB14, or control siRNAs. (*E*) Representative images of H441 cells migrating through Transwell inserts, transfected with TIAM1, TRIM28, and pan-PCDHβ/γ siRNAs, alone or in combination. (Scale bar, 400 μm.) The graph shows the average number of migrating cells normalized to control. (*F*) Model summarizing the role of TIAM1 in NSCLC. TIAM1 is part of the TRIM28-SETDB1 transcriptional corepressor complex that co-occupies genomic targets such as the clustered protocadherins, promoting H3K9me3 deposition. These epigenetic changes suppress gene transcription and lead to a more mesenchymal phenotype, promoting migration and invasion of NSCLC cells. Data are presented as mean ± SEM of three independent experiments. ns = nonsignificant, *P < 0.05 and ****P < 0.0001 (ANOVA).

Having demonstrated that TIAM1 promotes H3K9me3 and suppresses the expression of protocadherins and E-cadherin, we next performed Chromatin Immunoprecipitation sequencing (ChIP-seq) in H441 cells to determine the genomic loci occupied by TIAM1 and TRIM28 which are also rich in H3K9me3. Of the total TIAM1 peaks found by MACS peak caller, 41% (394/952) were found at gene promoters/transcriptional start sites (TSS) (Fig. 6*A*). Interestingly, 56% of the TIAM1 peaks (529/952) overlapped with H3K9me3 peaks (*SI Appendix*, Fig. S7*A*). Moreover, there was an overlap of peaks for TIAM1, TRIM28, and H3K9me3 (Fig. 6*B*), where 66% and 65% of the TRIM28 peaks overlapped with TIAM1 and H3K9me3 binding peaks, respectively (*SI Appendix*, Fig. S7*B*). This suggests that TIAM1 and TRIM28 co-occupy sites of heterochromatin rich in H3K9me3.

Next, we analyzed the TIAM1, TRIM28, and H3K9me3 occupancy at the clustered protocadherin locus on chromosome 5q31 and the E-cadherin gene on chromosome 16q22.1. The ChIP-seq analysis described above, performed on H441 cells without the addition of EMT inducer, revealed overlapping peaks for TIAM1, TRIM28, and H3K9me3 at two sites within the clustered protocadherin locus (Fig. 6*C*). Distinct narrow peaks for TIAM1 and TRIM28 were present upstream of the PCDH-α cluster and between the PCDH-β and PCDH-γ clusters. Broad peaks for H3K9me3 were present, decorating the chromatin up and downstream of the TIAM1-TRIM28 complex, occupying all PCDH genes (Fig. 6*C*). In contrast, we found that the CDH1 locus was not occupied by TIAM1 nor TRIM28, nor was it decorated by H3K9me3 (*SI Appendix*, Fig. S7*C*), consistent with abundant E-cadherin expression in H441 cells prior to treatment with EMT inducer (*SI Appendix*, Fig. S6 *A*, *D,* and *F*). Furthermore, TIAM1 depletion in H441 cells did not alter Snail and ZEB1 expression, suggesting that TIAM1 is not indirectly repressing E-cadherin through these repressive transcription factors (*SI Appendix*, Fig. S7 *D* and *E*). Interestingly, specific depletion of PCDHB9, but not PCDHB3 or PCDHB14, led to a reduction in CDH1 expression (Fig. 6*D* and *SI Appendix*, Fig. S7*F*). Taken together, these results indicate that nuclear TIAM1 is part of the TRIM28 transcriptional corepressor complex that directly suppresses the expression of protocadherins and likely regulates E-cadherin indirectly through PCDHB9.

Migration of lung epithelial cells is associated with EMT (45). Having demonstrated that TIAM1 and TRIM28 promote EMT via downregulation of E-cadherin, and that they directly repress protocadherins, which themselves can regulate EMT in cancer (46), we analyzed the functional significance of this regulation on NSCLC cell migration. Consistent with the data presented above, TIAM1 depletion significantly reduced the migration of H441 cells in response to TGF-β (Fig. 6*E* and *SI Appendix*, Fig. S8*A*). This was also the case for TRIM28 depletion using two different siRNAs (Fig. 6*E* and *SI Appendix*, Fig. S8*A*). Migration of H358 cells in response to wounding was also reduced following TIAM1 or TRIM28 depletion (*SI Appendix*, Fig. S8 *B* and *C*). To test whether decreased migration following TIAM1 or TRIM28 knockdown was due to upregulation of protocadherins, TIAM1, TRIM28, and the β and γ protocadherins were depleted using siRNAs in different combinations (Fig. 6*E* and *SI Appendix*, Fig. S8 *A–C*). Whereas TIAM1 or TRIM28 depletion led to a reduction in migration, pan-protocadherin-β/γ depletion either induced a migratory response or did not influence migration compared to control cells (Fig. 6*E* and *SI Appendix*, Fig. S8*B*). Importantly, the decrease in migration observed after TIAM1 or TRIM28 depletion was dramatically reversed by the simultaneous depletion of pan-protocadherin-β/γ (Fig. 6*E* and *SI Appendix*, Fig. S8*B*). To explore the association between protocadherins and NSCLC progression, Kaplan–Meier curves of overall survival in LUAD defined by protocadherin expression were generated using the Kaplan–Meier Plotter (47). High expression levels of PCDHB3, B9, and B14 predicted better survival in LUAD (*SI Appendix*, Fig. S8*D*). Taken together, these findings indicate that TIAM1, together with TRIM28, promotes the migration of NSCLC cells by suppressing the expression of protocadherins.

## Discussion

Here, we present evidence that nuclear TIAM1 promotes NSCLC cell migration by participating in a transcriptional repressor complex involving TRIM28 and SETDB1, that mediates the epigenetic silencing of cell–cell adhesion genes (Fig. 6*F*). This contrasts with what we have described previously in CRC, where nuclear TIAM1 suppresses migration by preventing the interaction of YAP/TAZ with TEAD transcription factors, inhibiting the expression of YAP/TAZ target genes (30). Interestingly, in NSCLC, TAZ was not identified in the dual proteomic screen as an interactor of nuclear TIAM1, nor was there an enrichment for the YAP conserved signature in TIAM1-depleted cells. This suggests that the role of nuclear TIAM1 in migration is dependent on the cellular context and may differ by cancer type. Importantly, we also found that nuclear TIAM1 levels were increased in advanced-stage NSCLC human tumors and correlated with poor survival of NSCLC patients, again in contrast to what we have previously observed in colorectal cancer (30).

In this study, we focused on the nuclear roles of TIAM1 in NSCLC. Nuclear functions for TIAM1 have been reported previously, but apart from our study on colorectal cancer mentioned above, nuclear TIAM1 has not been linked with cell migration. Specifically, it has previously been shown that nuclear TIAM1 in fibroblasts interacts with c-Myc, repressing its transcriptional activity and inhibiting c-Myc-mediated apoptosis (48). Moreover, in Th17 immune cells, nuclear TIAM1 was shown to interact with the transcription factor RAR-related orphan receptor gamma (RORγt) to stimulate the interleukin (IL)17 promoter (34). Additionally, we recently identified a nuclear role for TIAM1 in SCLC unrelated to changes in gene expression. We showed that TIAM1 interacts with and sequesters the orphan nuclear receptor Nur77 in the nucleus of SCLC cells preventing its translocation to the cytoplasm where it binds BCL2 inducing its proapoptotic conformational change (49). Therefore, both scaffolding and gene regulation functions for nuclear TIAM1 have been described; however, a link between TIAM1 and chromatin modification had not been made previously.

In this study, we found that in NSCLC cells, nuclear TIAM1 interacts with TRIM28 and SETDB1 and regulates chromatin modification to silence gene expression. TRIM28 has been described as a master regulator of gene expression, forming a complex with zinc finger transcription factors and SETDB1 to silence target genes (43). SETDB1 is known to mediate H3K9me3 deposition and heterochromatin formation (50). Interestingly, our results suggest that TIAM1 acts as a scaffold to bridge the interaction between TRIM28 and SETDB1. Our data also indicate that a complex including TIAM1 and TRIM28 suppresses the expression of protocadherins directly. SETDB1 has previously been shown to occupy the clustered PCDH locus in neuronal cells and depletion of SETDB1 led to a reduction in H3K9me3 methylation and PCDH expression (51). TRIM28 has also been shown to regulate the expression of PCDHB6 through repressive H3K9me3 marks in neurons in vivo (52). Interestingly, we show that the TIAM1-TRIM28 transcriptional corepressor complex regulates these cell–cell adhesion molecules in cancer. Moreover, our data indicate that the suppression of E-cadherin expression by TIAM1 is likely indirect, as TIAM1 and TRIM28 were not found at the CDH1 locus in H441 cells. In keeping with our findings, TRIM28 was previously shown to suppress the expression of E-cadherin in NSCLC cells, although occupancy at the CDH1 promoter was again not observed (42). Interestingly, we have shown that PCDHB9 regulates E-cadherin expression, suggesting that TIAM1 regulates E-cadherin expression indirectly through its effects on PCDHs. This is consistent with published findings showing that PCDHs regulate E-cadherin expression, but that the responsible PCDH may be cell type specific. For example, overexpression or depletion of PCDHB3 led to an up- or downregulation, respectively, of E-cadherin in CRC (53). It was shown that PCDHB3 promoted increased binding of the NF-κB subunit p50 to Snail and ZEB1 promoters, suppressing their transcription and their subsequent regulation of genes encoding cell–cell adhesion proteins, including E-cadherin (53). Furthermore, in gastric cancer, PCDHGA9 was shown to reduce SMAD2/3 nuclear translocation suppressing Snail expression, resulting in changes in cell–cell adhesion proteins, including E-cadherin (46). As we did not observe changes in transcription factors that control E-cadherin following TIAM1 depletion, there are likely alternative mechanisms for the regulation of E-cadherin by PCDHB9 in NSCLC.

We hypothesize that nuclear TIAM1 promotes the loss of epithelial architecture associated with high-grade NSCLC tumors through the downregulation of adhesion molecules, such as E-cadherin and protocadherins. This hypothesis is consistent with the increased nuclear TIAM1 we observed in late-stage NSCLC tumors, the negative association existing between protocadherin expression and survival of NSCLC patients, and the inverse correlation of nuclear TIAM1 with E-cadherin expression in NSCLC patients. The clustered protocadherins are the largest subfamily of cadherin proteins and display structural similarities to classical cadherins in possessing an intracellular domain, transmembrane domain, and six extracellular domains (compared to five in classical cadherins) (54, 55). While E-cadherin is an undisputed determinant of epithelial characteristics, protocadherins have been relatively underresearched. Despite known roles in neural development (56), evidence also suggests that protocadherins can mediate cell adhesion and migration. For example, expression of protocadherin-γ-A3 and protocadherin-γ-C3 induced calcium-dependent aggregation of HEK-293 cells and mouse fibroblasts (57). Moreover, overexpression or depletion of PCDHGA9 induced a decrease or increase, respectively, in the migration and invasion of gastric cancer cells (46), and PCDHB3 inhibited the migration of CRC cells (53). In further support of an antimigratory function, we now show that the decrease in migration observed after TIAM1 or TRIM28 depletion was dependent on protocadherins.

Overall, our data show that nuclear TIAM1 promotes the migration and invasion of NSCLC cells in vitro and in vivo, which would be anticipated to contribute to their increased metastatic potential. Interestingly, we demonstrated that the role of nuclear TIAM1 in promoting NSCLC cell migration depends on RAC1 activity. Previous findings in other cancers have demonstrated that TIAM1-RAC1 signaling suppresses cell migration (27, 29, 30), which contrasts with our findings now in NSCLC. This highlights how the effects of signaling modules depend on the signaling network in which they are embedded, as well as the importance of establishing this context for each cancer type. In NSCLC, therefore, targeting TIAM1-RAC1 signaling would be a viable strategy for suppressing disease progression. Moreover, TRIM28 expression is increased in NSCLC and may be used as a prognostic marker (58, 59). The results of our study suggest nuclear TIAM1 and protocadherin expression could also be used as prognostic markers. An index based on TRIM28 expression, nuclear TIAM1 intensity, and protocadherin expression could therefore provide clearer prognostic information. Furthermore, due to the role of TIAM1 in promoting a more mesenchymal phenotype, and given the role of EMT in promoting drug resistance (11), it may be of importance in therapeutic resistance of NSCLC.

## Materials and Methods

Details on methods used are provided in *SI Appendix*, covering Immunohistochemical Analysis of Human LUAD Clinical specimens, Cell Culture, Cell Transfection, Nuclear Fractionation, Western Blotting, Cell Imaging, Scratch Wound, and Transwell Migration Assays, Viability Assays, Generation of Stable Cells Expressing TIAM1 Constructs, Zebrafish Embryo Xenografts, Immunoprecipitation, BioID, Mass Spectrometry, Chromatin Fractionation, Induction of EMT, RNA-Seq and Bioinformatics Analysis, qRT-PCR, ChIP-Seq and Bioinformatics Analysis, Outcome Analysis, and Statistical Analysis.

## Supplementary Material

Appendix 01 (PDF)

Dataset S01 (XLSX)

## Data Availability

The RNA-seq and ChIP-seq data reported in this paper have been deposited to National Center for Biotechnology Information Gene Expession Omnibus (NCBI GEO) (http://www.ncbi.nlm.nih.gov/geo) under accession number GSE219030 (60). All other data are included in the manuscript and/or supporting information.
