# Supplementary material for: A TIAM1-TRIM28 complex mediates epigenetic silencing of protocadherins to promote migration of lung cancer cells
Source: Proc Natl Acad Sci U S A. 2023 Sep 25;120(40):e2300489120. doi: 10.1073/pnas.2300489120 (PMC10556593; doi:10.1073/pnas.2300489120)
Supplement: Supplementary file 1 — Appendix 01 (PDF) [file pnas.2300489120.sapp.pdf]

PNAS

Supporting Information for

A TIAM1-TRIM28 complex mediates epigenetic silencing of protocadherins to promote migration of lung cancer cells

Lucy Ginn, Joe Maltas, Martin J. Baker, Anshuman Chaturvedi, Leah Wilson, Ryan Guilbert, Fabio M.R. Amaral, Lynsey Priest, Holly Mole, Fiona Blackhall, Zoi Diamantopoulou, Tim C.P. Somervaille, Adam Hurlstone and Angeliki Malliri

Corresponding author: Angeliki Malliri

Email: angeliki.malliri@cruk.manchester.ac.uk

This PDF file includes:

Materials and methods

Figures S1 to S8

Tables S2 to S4

Supporting references

Other supporting materials for this manuscript include the following:

SI Table 1, provided as an Excel file

Materials and methods

Immunohistochemical analysis of human LUAD clinical specimens. Immunohistochemistry for TIAM1 and E-cadherin was performed with a standard avidin-biotin complex method (ABC) on stage I – IV adenocarcinoma FFPE samples. Samples from stage I (n=20), II (n=20) and III (n=13) patients were collated within a TMA sourced via the ETOP (European Thoracic Oncology Platform) Lungscape study (REC Ref 12/LO/0235) (1). Samples were sourced and assessed from the Pathology Department, Wythenshawe Hospital, Manchester Foundation Trust and were de-identified prior to use in this study. The design and construction of the TMA was in line with ETOP Lungscape ALK-1 Pathology Protocol. In brief, two FFPE blocks were identified from each of the selected and approved patient cases that met the inclusion criteria and quality assurance parameters of the study. Two, 0.6mm cores were sampled from identified regions of tumour for each block. A total of 4 cores of tumour tissue were included for each case. Twenty-eight stage IV diagnostic FFPE samples, along with their clinical pathological data, were also sourced from the ChemoRes (Molecular mechanisms underlying chemotherapy resistance, therapeutic escape, efficacy and toxicity) study (REC Ref 07/H1014/96). One full FFPE block was provided for each patient. Following completion of the immunohistochemistry, the slides were examined blindy using HALO AI™ Image Analysis Software and by a histopathologist. The intensity of immunostaining (negative 0, weak 1, moderate 2, strong 3) for TIAM1 and its subcellular localisation was assessed. H-scores were generated by HALO AI™ software based on tumour area and percentage of cells with each staining intensity score (0-3). Tumour area was classified using HALO AI™ based on TIAM1 IHC. Matched serial sections for E-cadherin and TIAM1 staining were linked using HALO AI™ software. E-cadherin staining intensity was assessed within matched tumour areas to ensure the same area was quantified for TIAM1 and E-cadherin staining. For histopathological assessment, overall TIAM1 and E-cadherin staining intensity scores for each patient were used. Correlation analysis was performed per sample for the stage I-IV patients. Statistical analysis including survival analysis was performed using GraphPad Prism.

Cell culture. All cell lines were cultured at 37ºC with 5% CO_2. H1299, H441 and H358 were cultured in RPMI-1640 Medium (Sigma-Aldrich, #21875034), supplemented with 10% FBS and 1% penicillin/streptomycin (Gibco, #15140122). Phoenix-GP, HEK293 and LentiX-293T cells were cultured in DMEM supplemented with 10% FBS and 1% penicillin/streptomycin. For antibiotic selection, culture medium of cell lines harbouring the pRetroX-Tight-Pur plasmids was supplemented with 750μg/ml G418 (Sigma-Aldrich, #10131035) and 2 μg/ml Puromycin (Sigma-Aldrich, #P8833) and LT3GEPIR plasmids with 2 μg/ml Puromycin. Cells were regularly authenticated and checked for mycoplasma infection through in-house facilities.

Cell transfection. Transfection of plasmid DNA into Phoenix-GP cells was performed using TransIT-®-LT1 transfection reagent (Mirus, #MIR2300) according to manufacturer's instructions. Transfection of plasmid DNA into HEK293 and LentiX-293T cells was performed using Fugene® HD transfection

reagent (Promega, #E2311) according to manufacturer's instructions. A list of DNA constructs used in this study are outlined in Table S2. Reverse transfection of siRNA oligonucleotides was performed using Lipofectamine® RNAiMAX Reagent (Thermo, #13778150) according to manufacturer's instructions. A list of siRNA or control oligonucleotide sequences (MWG Operon) designed against human TIAM1, TRIM28, pan-PCDH-β/γ or individual PCDHs are outlined in Table S3.

Nuclear fractionation. Cells were washed with PBS before being incubated in hypotonic buffer (20mM Tris-HCL (pH 7.4); 10mM NaCL; 3mM $MgCl_2$) containing phosphatase and protease inhibitor cocktails (Sigma, #4906837001, #P8340) on ice for 15 minutes. 10% NP40 was then added and samples were vortexed for 10 seconds. Samples were centrifuged at 0.8 x g for 10 minutes at 4°C. The supernatant containing the soluble cytoplasmic proteins was collected. The nuclear pellet was resuspended and incubated in Cell Extraction Buffer (Thermo, #FNN0011), containing phosphatase and protease inhibitor cocktails (Sigma), on ice for 30 minutes, vortexing at 10-minute intervals. The samples were then centrifuged at 14,000 x g for 30 minutes at 4°C. The supernatant containing the soluble nuclear proteins was collected. Samples were immediately used for immunoprecipitation or stored at -80°C for later protein analysis.

Western blotting. Cells were lysed in RIPA buffer (Sigma, #R0278) or Laemmli buffer (Bio-Rad, #1610747) and protein extracts were collected, resolved by SDS-PAGE and transferred to nylon membranes (Merck, #IPVH00010) using standard techniques. Membranes containing the separated proteins were blocked in 10% milk-PBST and then incubated with primary antibodies overnight. Membranes were then incubated with HRP-conjugated secondary antibodies to enable detection and measurement of the levels of primary antibodies bound to the proteins on the membrane, using X-ray film or a developer machine (BioRad Chemidoc). Primary and secondary antibodies used for Western blotting are outlined in Table S4.

Cell imaging. Cells were grown on coverslips and fixed with 3.7% Formaldehyde (v/v). Coverslips were washed and either mounted immediately onto glass slides using ProLong Gold Antifade Mountant with DAPI (Thermo, #P36935) for GFP fluorescence analysis or otherwise left overnight at 4ºC before processing the next day for immunofluorescence. In this case, cells were permeabilised in 0.2% Triton (v/v), then blocked with 1% BSA before successive incubation with primary and then secondary antibodies. Finally, coverslips were mounted and processed as above. Antibodies used for immunofluorescence are listed in Table S4. Fluorescence images were obtained using either the low light microscope system, based on a Zeiss Axiovert 200M enclosed in a full environmental chamber (Solent Scientific), or acquired using a Zeiss Observer microscope equipped with a Zeiss LSM 880 scan head with the AiryScan detector. Bright-field images were obtained using the Zeiss Axiovert 25C inverted microscope. Captured images were processed using ImageJ software.

Scratch wound migration assays. Migration assays were performed using 96-well Incucyte® Scratch Wound Assay according to manufacturer's instructions. Cells treated with indicated siRNAs were seeded into a 96-well image lock plate as a confluent cell monolayer in 10% FBS (H358) or 2.5% FBS (H441) media. Once cells had attached to the plate, a wound was generated, and images were taken every hour for 48 hours (H358) or 12 hours (H441) using the Incucyte® Zoom system. Images were analysed using Incucyte ® Software for wound confluence (confluence of cells within the wound) as a measure of cell migration. Values were background subtracted to account for any differences in initial wound size (t=0).

Transwell migration and invasion assays. Cells were treated with siRNA oligonucleotides and doxycycline as indicated, for 72 hours. Cells were then starved in 0.2% FBS media and seeded onto transwells with 8µm pores (Costar, #3422 & Starstedt TC inserts, #83.3932.800). For invasion assays, 50 µL 50 µg/mL collagen I was left to set on membranes for 2 hours and excess removed. Cells migrated or invaded for 16 hours towards 10 ng/µL TGFβ or 10 ng/µL HGF (Sigma, #H8541) (0.2% FBS). Unless otherwise indicated, TGFβ was used as a chemoattractant in transwell migration assays. Migrated/invaded cells were fixed in 0.5% crystal violet (in methanol) for 20 minutes. Filters were washed in ddH_2O and non-migrated/invaded cells were scraped off the upper side of the filters. Relative migration/invasion was assessed on ImageJ, where crystal violet staining pixel area was quantified as a measure of migrating/invading cells. For migration experiments involving NSC23766 (Tocris, #2161), cells were treated with the drug for 24 hours prior to transwell seeding and viability was assessed for the duration of cell migration through transwells.

Viability assays. For proliferation assays, cells were reverse transfected and seeded in sets of 3 or 4 plates and viability was assayed from day 0 to day 5 using cell Titre Glo® 2D (Promega, cat# G7572) according to manufacturer's guidelines in a 96 well format. Luminescence was measured as an indicator of cell viability (Promega GloMax Multimode detection system). Proliferation over time was assessed by percentage growth (% change in viability).

Generation of H1299, H441 and H358 cells inducibly expressing different TIAM1 constructs. The NES-TIAM1 plasmid was constructed by inserting the NES of MAPKK (MNLVDLQKKLEELELDEQQ) (previously described in (2)) into pcDNA3 FL-TIAM1, which was subsequently cloned into pRetroXtight. Phoenix-GP cells were transfected with pRetroXtight TIAM1 constructs, and retroviral transduction was performed as previously described (3). shRNA sequences were designed as previously reported in (4), then cloned into LT3GEPIR and lentiviral particles were produced in LentiX-293T cells. Transfections

were followed by appropriate antibiotic selection and inducible expression was controlled by the addition of doxycycline to cells.

Zebrafish embryo xenografts. *Nacre* zebrafish (Danio rerio) were maintained and bred by the University of Manchester Biological Services Facility. Experiments were approved by the ethical review body of the University of Manchester and performed according to the United Kingdom Animals (Scientific Procedures) Act 1986. Xenografts were generated from H441 cells expressing doxycycline-inducible TIAM1-targeting or control shRNAs along with GFP. Cells were pre-treated with or without 100 ng/ml doxycycline for 24 hours followed by staining with 20 μM Cell Trace Violet (Thermo, #C34557) for 30 minutes. Stained cells were dissociated and resuspended at $2x10^7$ cells/ml in 2% polyvinylpyrrolidinone K60 solution (Sigma, #81430) in DPBS (w/o Mg^{2+} and Ca^{2+}) and kept on ice until injection. 48-hour post-fertilisation zebrafish embryos were anesthetised in 0.16 mg/ml MS-222 (Sigma, # A5040) and cells injected into the pericardial cavity using a microinjection system with pulled glass capillary needles. Following injection, embryos were screened for the presence of Cell Trace Violet fluorescence in the pericardial cavity and retained embryos incubated with or without 1 μg/ml doxycycline as appropriate. Embryos were euthanised 5 days post fertilisation in 0.4 mg/ml MS-222 for 15 minutes and fixed with 4% formaldehyde in PBS for 24 hours at 4 °C. Fixed embryos were mounted in 1.5% low melt agarose and stacked images of the pericardial cavity obtained using a Leica SP8 upright confocal capturing GFP and Cell Trace Violet fluorescence. The GFP channel also captured autofluorescence from the embryo tissue allowing identification of the pericardial cavity boundaries and subsequent quantification of the number of cells that had invaded into the tissue surrounding the cavity (Figure S2F). Cells were considered to be invading if more than 50% of the cell had left the pericardial cavity and, if treated with doxycycline, had detectable GFP expression. The number of invaded cells per embryo were compared between untreated and doxycycline-treated for each of two TIAM1 targeting shRNA lines and one control shRNA line. Relative invasion was calculated using the formula: Number of cells invaded in an embryo/Mean number of cells invaded in -Dox for the cell line.

Immunoprecipitation. Protein G beads were pre-blocked with 5% BSA and cell fractionation or total samples [in IP lysis buffer (50 mM Tris-HCl pH 7.5, 150 mM NaCl, 1% (v/v) Triton-X-100, 10% (v/v) glycerol, 2 mM EDTA, 25 mM NaF, and 2 mM NaH2PO4)] were pre-cleared using IgG-conjugated Protein G beads. Samples were then incubated with beads conjugated to anti-TIAM1, anti-TRIM28 or anti-FLAG antibodies for 2 hours at 4ºC. Beads were then washed 5 times with IP lysis buffer and eluted with 20 μl 2 x SDS loading buffer. Samples were then processed for mass spectrometry or western blot analysis.

BioID. Cells were grown in low biotin medium (DMEM supplemented with 10% FBS and 1% penicillin/streptomycin) and supplemented with 50 μM biotin from 20 x biotin supplement stock for 24

hours. Protein was then extracted (as described above) or cells were fixed and immunofluorescence was performed (as described above). For isolation of biotinylated proteins, lysates were incubated with pre-washed MagReSyn Streptavidin Beads (ReSyn Biosciences) overnight at 4ºC. Beads were then washed twice with BioID wash buffer 1 (2% SDS), once with BioID wash buffer 2 [0.1% sodium deoxycholate; 1% Triton X-100; 500 mM NaCl; 1 mM EDTA; 50 mM Hepes (pH 7.4)] and once with BioID wash buffer 3 [250 mM LiCl; 0.5% NP-40; 0.5% deoxycholate; 1 mM EDTA; 10 mM Tris (pH 8.1)]. Proteins were eluted with 2 x SDS loading buffer saturated with biotin.

Mass spectrometry. Samples were analysed by the mass spectrometry in-house facility. In brief, coomassie-stained gel bands were de-stained by three incubations with 200 mM ammonium bicarbonate, 40% acetonitrile (v/v) at 37°C. Gel bands were then dehydrated by addition of acetonitrile. Gel bands were then rehydrated by addition of high-performance liquid chromatography (HPLC) grade water. The dehydration-rehydration cycle was performed a total of three times followed by a single dehydration stage with acetonitrile. Finally, the gel pieces were dried in a vacuum centrifuge. Gel bands were re-swollen by addition of 40 mM ammonium bicarbonate, 9 % acetonitrile (v/v) and 300 ng sequencing grade trypsin. Gel bands were then covered by addition of 40 mM ammonium bicarbonate, 9 % acetonitrile (v/v) followed by incubation at 37°C. Gel digests were acidified by addition of trifluoroacetic acid (TFA) to a final concentration of 0.1%. Digest supernatant was removed and dried. Peptides were resuspended in 0.1% TFA for injection. Nano-scale liquid chromatographic tandem mass spectrometry (nLC-MS/MS) was performed. Peptides were loaded directly onto a 25 cm long, 75 µm internal diameter, 2 µm C18 EasySpray column in 1 % acetonitrile, 0.1% formic acid (v/v) at a flow rate of 200 nl per minute using an RSLC nano HPLC system (Thermo). Peptides were separated with a gradient of 1 to 22% acetonitrile (v/v), 0.1% formic acid at a flow rate of 200 nl per minute. The nLC effluent was sprayed directly into the Orbitrap Fusion mass spectrometer (Thermo) at a voltage offset of 2.3KV. The mass spectrometer was performed at a nominal resolution of 1200,000 (at m/z 200) in the Orbitrap analyser between m/z range of 350-1000 with a target value of 2e5 ions and a maximum fill time of 50 milliseconds. The second stage of MS (MS2) was performed using higher-energy collisional dissociation (HCD) at a normalised collision energy of 28% with a target value of 1e4 ions and a maximum fill time of 50 milliseconds per MS2. Product ion spectra were collected in the linear ion trap using a rapid scan rate. Dynamic exclusion was enabled to prevent the selection of a formally targeted ion for a total of 6 seconds. Results were analysed on Scaffold 4 proteome software.

Chromatin fractionation. Chromatin fractions were isolated using the Subcellular Protein Fractionation Kit (Thermo Scientific^{TM}, #78840). Cells were washed with PBS before being incubated in Cytoplasmic Extraction Buffer (Thermo) for 10 minutes on ice. After a 5 min centrifugation at 500g, the cytoplasmic fraction was collected. The pellet was resuspended in Membrane Extraction Buffer (Thermo) for 10 min on ice. After a 5 min centrifugation at 3000 x g, the membrane fraction was collected. The pellet was resuspended in Nuclear Extraction Buffer (Thermo) for 30 min on ice. After a 5 min centrifugation at

5000 x g, the nuclear soluble fraction was collected. The pellet was resuspended in Nuclear Extraction Buffer containing $CaCl_2$ and Micrococcal Nuclease (Thermo) for 15 minutes at room temperature. After a 5 min centrifugation at 16,000 x g, the chromatin-bound nuclear fraction was collected. All buffers contained a protease inhibitor cocktail (Sigma).

Induction of EMT. H441 and H358 cells were treated with an EMT inducer cocktail from a 100 x stock (Recombinant human Wnt-5a, recombinant human TGF-β1, anti-human E-cadherin, anti-human sFRP-1, anti-human Dkk-1) (R&D Systems, #CCM017) for 5 days.

RNA-sequencing. RNA was extracted from cells using the RNeasy Kit (Qiagen, #74104) following the instructions of the manufacturer. Indexed PolyA libraries were prepared using 200 ng of total RNA and 14 cycles of amplification with the Agilent SureSelect Strand Specific RNA Library Prep Kit for Illumina Sequencing (Agilent, # G9691B). Libraries were quantified by qPCR using a Kapa Library Quantification Kit for Illumina sequencing platforms (Roche, # 07960336001). Single-read 75bp sequencing was carried out by clustering 2.0 pM of the pooled libraries on a NextSeq 500 sequencer (Illumina Inc.).

RNA-sequencing bioinformatics analysis. RNA-sequencing reads were quality-checked and aligned to the human genome assembly (GRCh38) using the STAR aligner v2.5.1b with the default parameter settings. Mapped data were converted to gene-level integer read counts using featureCounts and the Ensemble GTF annotation (Homo_sapiens.GRCh38.85.gtf). Differential expression (DE) was evaluated comparing the gene level integer read count data for between patient/sample groups using the DESeq2.v 1.26.0 Bioconductor package. P-values were adjusted using the Benjamini and Hochberg approach for controlling the false discovery rate. Genes with an adjusted P-value <=0.05 (FDR < =0.05) between two groups were considered to be differentially expressed.

Quantitative reverse transcription polymerase chain reaction (RT-qPCR). RNA was extracted from cells using the RNeasy Kit (Qiagen) following the instructions of the manufacturer. RNA was reverse transcribed to cDNA using the Omniscript RT Kit (Qiagen, 205113) or UltraScript 2.0 cDNA synthesis kit (PCR-BIO, #PB30.31-10) according to the manufacturer’s instructions. qPCR was performed in triplicate in a 20 μl reaction mixture containing 10 μl of 2× TaqMan® master mix, 0.5 μM of each of the primers and cDNA. The reaction mixture without a template was run as a control. ACTB and GAPDH expression levels were used to normalise for differences in RNA input. Primers for ACTB, GAPDH, E-cadherin, TRIM28 and TIAM1 were designed online using the Universal Probe Library Assay Design Center. Primers for PCDHB3, PCDHB9, PCDHB14 and pan-PCDH-γ were designed online using the Universal Probe Library Assay Design Center. Primers used for RT-qPCR are listed in Table S3.

Chromatin immunoprecipitation sequencing (ChIP-seq). Chromatin immunoprecipitation was performed using the SimpleChIP® Enzymatic Chromatin IP Kit (Magnetic Beads) (Cell Signalling Technology, #9003) following the instructions of the manufacturer. 10 µg of chromatin was incubated overnight with 1.6 µg of the sheep anti-TIAM1, rabbit anti-TRIM28 or rabbit anti-H3K9me3 antibody. ChIP-seq libraries were prepared using 1 ng of ChIP DNA and 12 cycles of amplification with the NEBNext Ultra II DNA Library Prep Kit for Illumina (New England BioLabs, #E7645L). Libraries were quantified by qPCR using a KAPA Library Quantification Kit for Illumina sequencing platforms (Roche, #07960336001). Paired-end 75bp sequencing was carried out by clustering 1.7 pM of the pooled libraries on a NextSeq 500 sequencer (Illumina Inc.). Antibodies used for ChIP are listed in Table S4.

ChIP-seq bioinformatics analysis. Sequencing reads from ChIP and input samples were quality-checked using FASTQC. All bases with a Phred quality score <= 20 and any adapter sequences present in the data were removed using FASTX-Toolkit. The cleaned and trimmed FASTQ files from previous steps were mapped to the hg19 reference assembly using bowtie2.v2.2.1. The resulting alignment SAM files were processed using samtools v1.3.1 to remove unmapped reads, and to retain reads with a mapping quality >= 20. Finally, the SAM files were converted into BAM files and indexed. Peak calls were generated from the alignment results (BAM files) from MACS2 v2.1.2 (with –keep-dup 1 -q 0.01 parameters for narrow peaks and with --broad-cutoff 0.1 for broad peaks) for peak calling to identify the ChIP signal enriched genomic regions. Homer v4.10 was used to annotate the significant peaks and TSS/Genebody profiles were generated by ngsplot v2.61. Homer Known Motif Analysis in R with default parameters was performed as indicated.

Outcome analysis. The KM plotter tool (5) (kmplot.com) was used to evaluate the survival of LUAD patients split by median expression of specific PCDH genes PCDHB3, B9 and B14. The database contains 719 LUAD patient samples. HR and p values for log-rank test are automatically generated for each analysis and are displayed on each plot.

Statistical analysis. All error bars shown on graphs represent ± standard error of the mean. The specific statistical tests used are indicated in the figure legends alongside the p values and were carried out using GraphPad Prism. For comparison between two conditions, statistical tests can be assumed to be a two-tailed Student's t-test. For multiple comparisons, ANOVA was used.

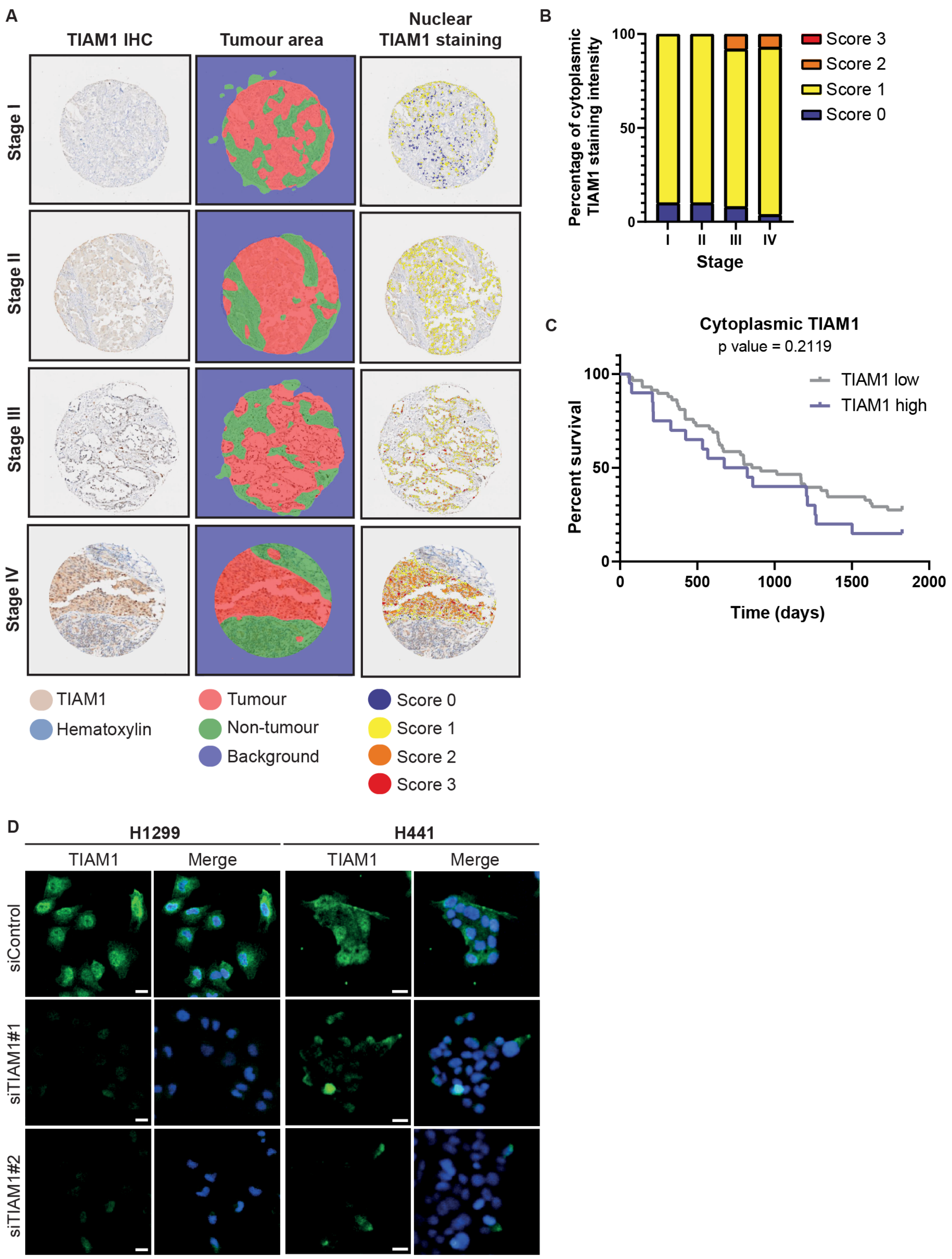

Fig. S1. Abundance of nuclear, but not cytoplasmic, TIAM1 correlates with NSCLC progression

(A) Representative images from the HALO AI TIAM1 IHC analysis pipeline. Samples stained for TIAM1 were used to classify tumour areas of tissue. Analysers were generated to detect nuclear TIAM1 staining intensity (scores 0-3) within the tumour areas only. (B) Histopathologist analysis of cytoplasmic TIAM1 staining intensity in stage I-IV patients. (C) Kaplan–Meier plot comparing the 5-year survival of patients with the highest quartile of cytoplasmic TIAM1 expression *vs.* the other three quartiles (p-value shown for Mantel-Cox test). (D) Representative immunofluorescence images of H1299 and H441 cells transfected with TIAM1 or control siRNAs, stained for DNA with DAPI (blue) and for TIAM1 (green) with a merged overlay. Scale bar= 20 μm.

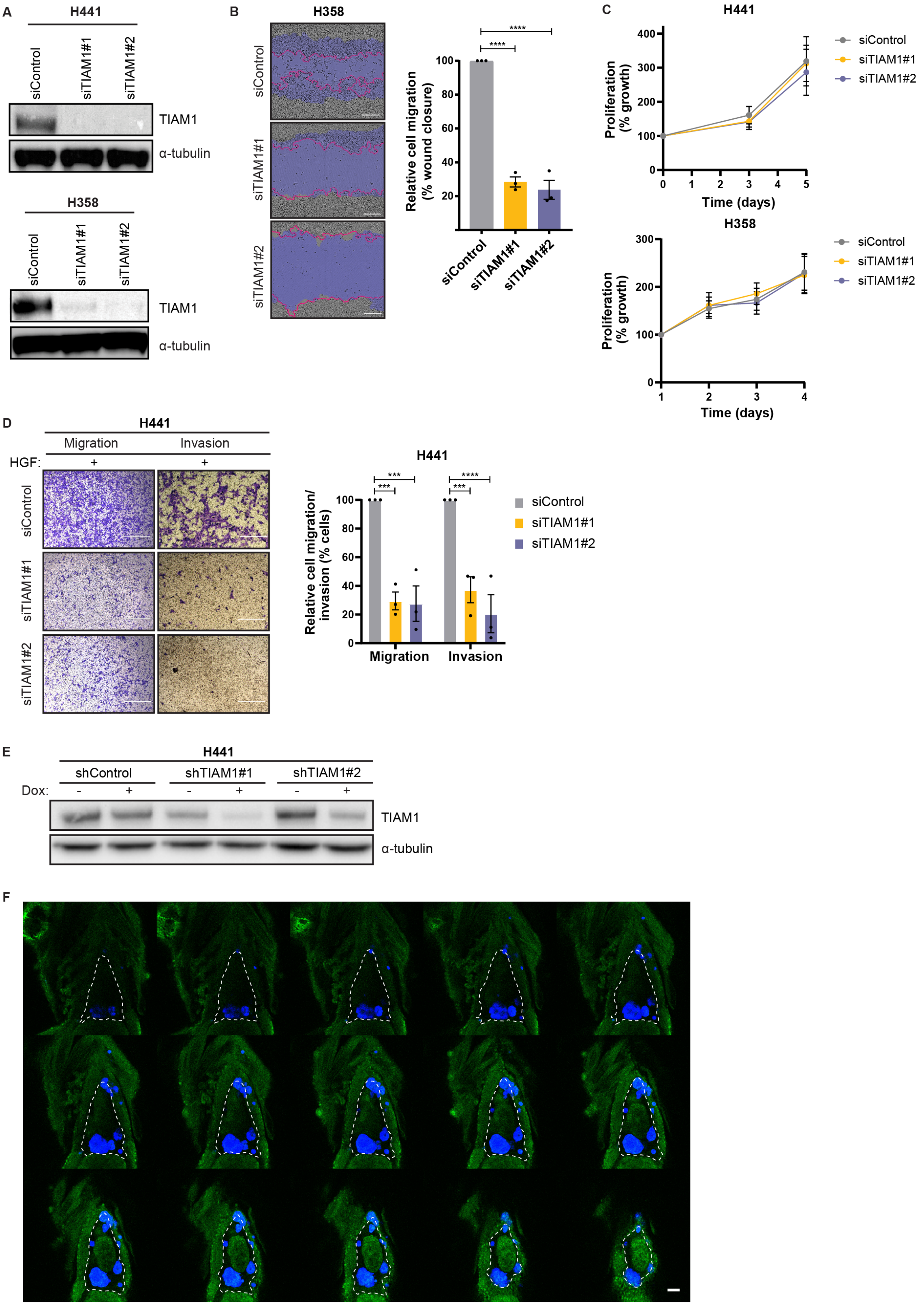

Fig. S2. TIAM1 depletion reduces NSCLC cell migration and invasion

(A) Western blot analysis of TIAM1 levels in H441 and H358 cells transfected with TIAM1 or control siRNAs used in migration assays. (B) Representative images of H358 cells transfected with TIAM1 or control siRNAs migrating through a wound. Scale bar, 200 μm. Graph shows percentage wound closure relative to initial wound size. (C) Graphs showing proliferation over time of H441 and H358 cells transfected with TIAM1 or control siRNAs. (D) Representative images of H441 cells transfected with TIAM1 or control siRNAs migrating or invading through

collagen I coated transwell inserts. 10ng/ml HGF was used as a chemoattractant. Scale bar= 1000 µm (migration), 400µm (invasion). Graphs show percentage (%) of migrating or invading cells normalised to control. (E) Western blot analysis of TIAM1 levels in H441 cells expressing doxycycline-inducible control shRNA or shRNAs targeting TIAM1, used in zebrafish studies. (F) Representative montage of z-stack slices for an untreated shTIAM1#2 xenograft with the pericardial cavity indicated. Cell Trace Violet stained H441 cells are shown in blue and tissue autofluorescence in green showing the boundary of the pericardial cavity and the heart in the centre of the cavity. Scale bar, 50 µm. Data presented as mean ± SEM of three independent experiments. *** $p<0.001$, **** $p<0.0001$ (ANOVA).

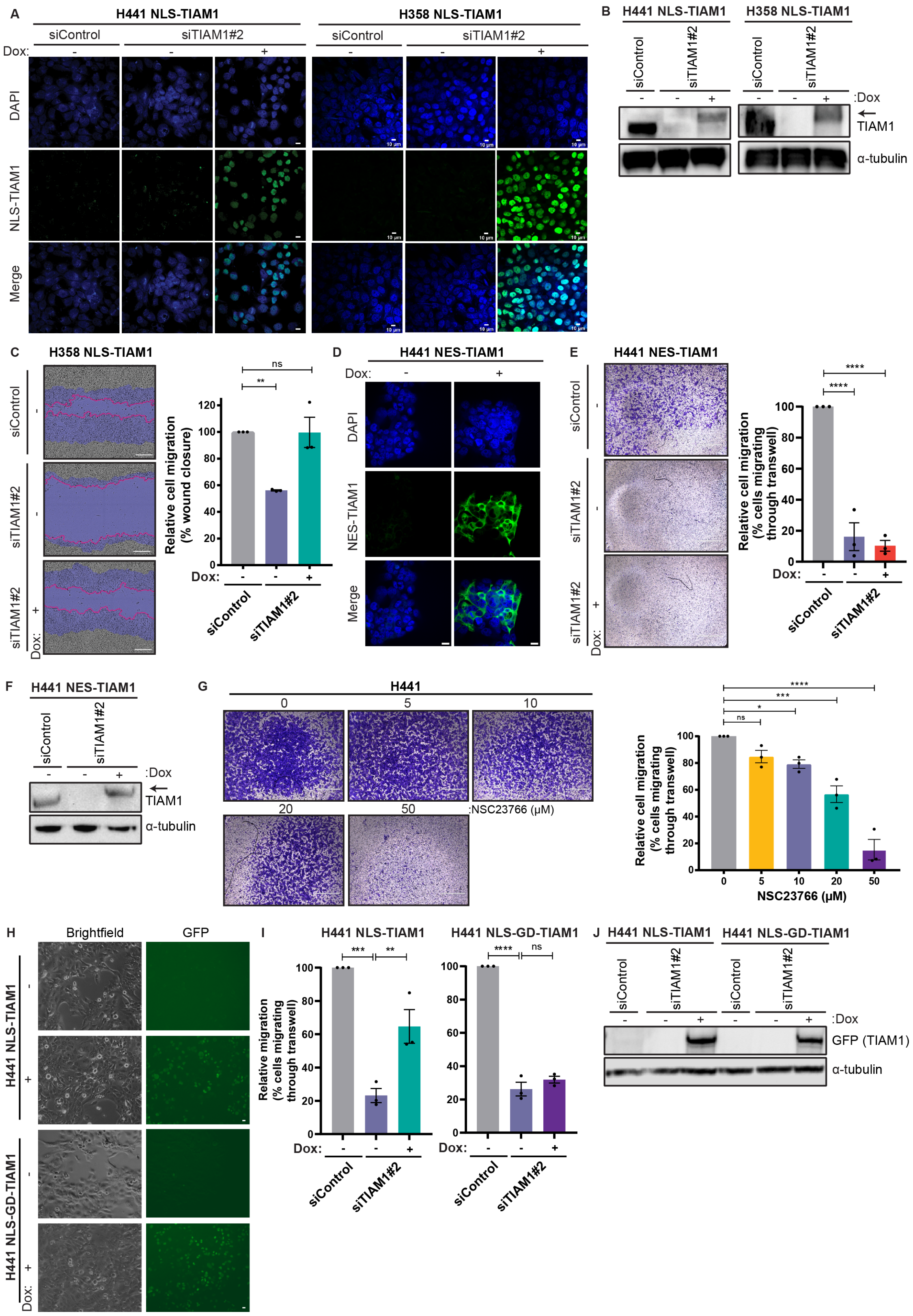

Fig. S3. Nuclear TIAM1 and RAC1 promote NSCLC migration

(A) Fluorescence images of H441 and H358 cells transfected with TIAM1 or control siRNAs stained with DAPI (blue) or showing tagged protein fluorescence (green) after induction of NLS-TIAM1 expression with doxycycline (+). Scale bar, 10 μm. (B) Western blot analysis of TIAM1 levels in H441 and H358 cells used for migration assays,

transfected with TIAM1 or control siRNAs and inducibly expressing NLS-TIAM1 (arrow), following the addition of doxycycline. (C) Representative images of migrating H358 cells transfected with TIAM1 or control siRNAs and inducibly expressing NLS-TIAM1 following the addition of doxycycline. Scale bar, 200 μm. Graph shows percentage wound closure relative to initial wound size, where control cell migration is set to 100%. (D) Fluorescence images of H441 cells after induction of NES-TIAM1 expression with doxycycline (+). Scale bar, 24 μm. (E) Representative images of H441 cells migrating through transwell inserts, transfected with TIAM1 or control siRNAs and inducibly expressing NES-TIAM1 following the addition of doxycycline. Scale bar, 1000 μm. Graph shows average percentage of migrating cells where control cell migration is set to 100%. (F) Western blot analysis of TIAM1 levels in H441 cells inducibly expressing NES-TIAM1 (arrow) used in E. (G) Representative images of H441 cells migrating through transwell inserts after treatment with an increasing dose of the RAC1 inhibitor NSC23766. Scale bar, 1000 μm. Graph shows average percentage of migrating cells normalised to viability and then to DMSO control. (H) Brightfield or GFP fluorescence images of H441 cells inducibly expressing NLS- or NLS-GD-TIAM1. Scale bar, 30 μm. (I) Graph shows average percentage of H441 cells transfected with TIAM1 or control siRNAs and inducibly expressing NLS- or NLS-GD-TIAM1 following the addition of doxycycline, migrating through transwell inserts. Scale bar, 1000 μm. (J) Western blot analysis of H441 cells transfected with TIAM1 or control siRNAs and inducibly expressing NLS- or NLS-GD-TIAM1-GFP. Data presented as mean ± SEM of three independent experiments. ns = non-significant, * $p<0.05$, ** $p<0.01$, *** $p<0.001$, **** $p<0.0001$ (ANOVA).

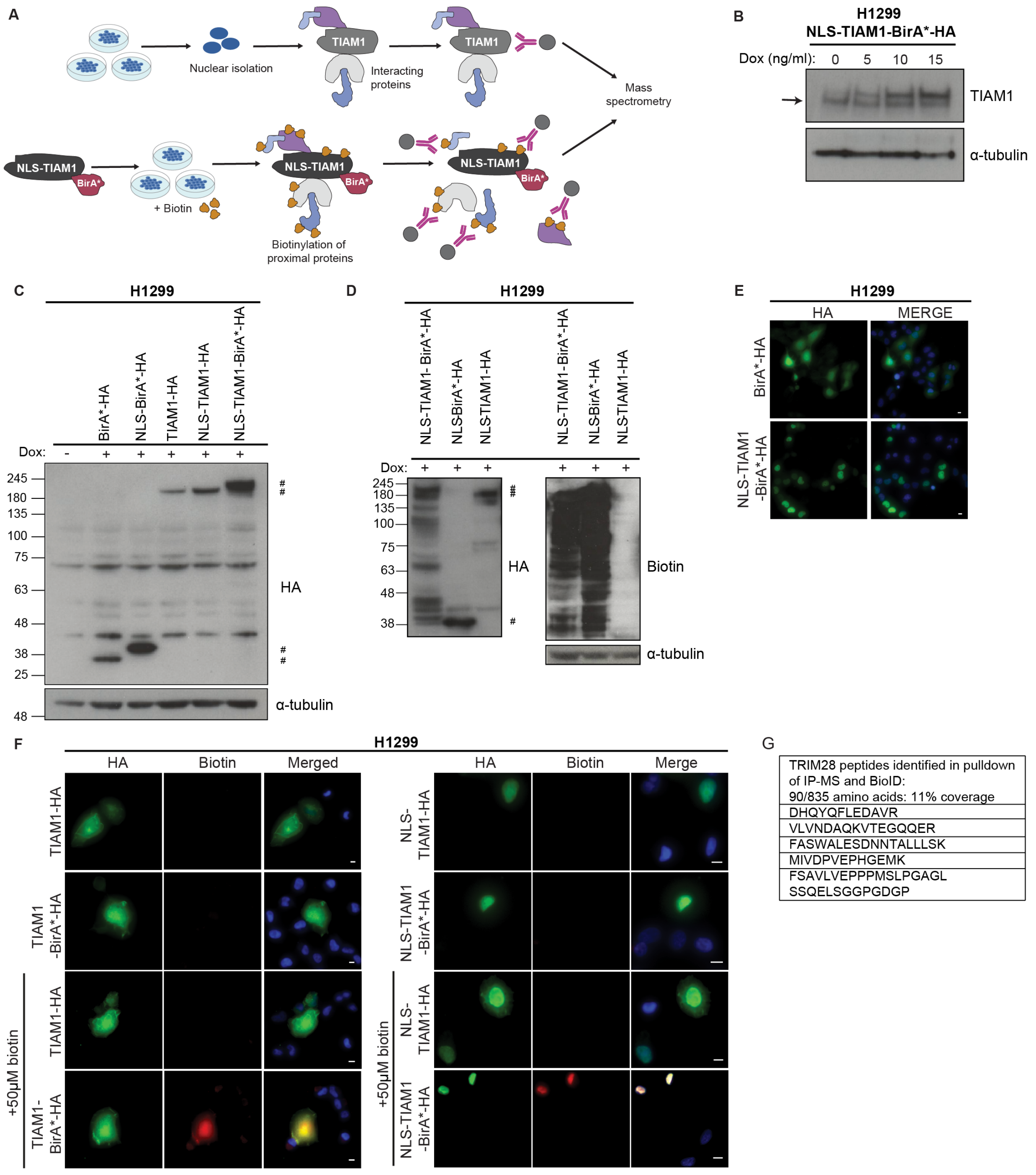

Fig. S4. Identification of the TIAM1-TRIM28 interaction

(A) Schematic illustration of the dual proteomic screen used to identify nuclear interactors of TIAM1. (B) Western blot analysis of H1299 cells inducibly expressing NLS-TIAM1-BirA*-HA following the addition of various concentrations of doxycycline (from 5 ng/ml to 15 ng/ml). Arrow indicates endogenous TIAM1. (C) Western blot analysis of the BioID and TIAM1 constructs after the addition of doxycycline (+). Constructs, detected with an anti-HA antibody, are indicated by #. Parental H1299 cells were included as a negative control (-). (D) Western blot analysis of biotinylation in H1299 NLS-TIAM1-BirA*-HA and H1299 NLS-BirA*-HA cells (as well as NLS-TIAM1-HA cells as a control) supplemented with 50 µM biotin. Constructs, detected with an anti-HA antibody, are indicated by #. (E) Immunofluorescence images of H1299 cells expressing BirA*-HA or NLS-TIAM1-BirA*-HA stained with DAPI (blue) and for HA (green) with a merged overlay. Scale bar, 10 µm. (F) Immunofluorescence images of H1299 TIAM1-HA, H1299 TIAM1-BirA*-HA, H1299 NLS-TIAM1-HA and H1299 NLS-TIAM1-BirA*-HA cells in the absence

or presence of biotin, stained with DAPI (blue), and for HA (green) and biotin (red) with a merged overlay. Scale bar, 10 μm. (G) Peptide sequences of TRIM28 identified exclusively in the pulldown of nuclear TIAM1 by IP-MS and NLS-TIAM1-BirA*-HA by BioID and not in control samples. Amino acid count and % coverage are shown.

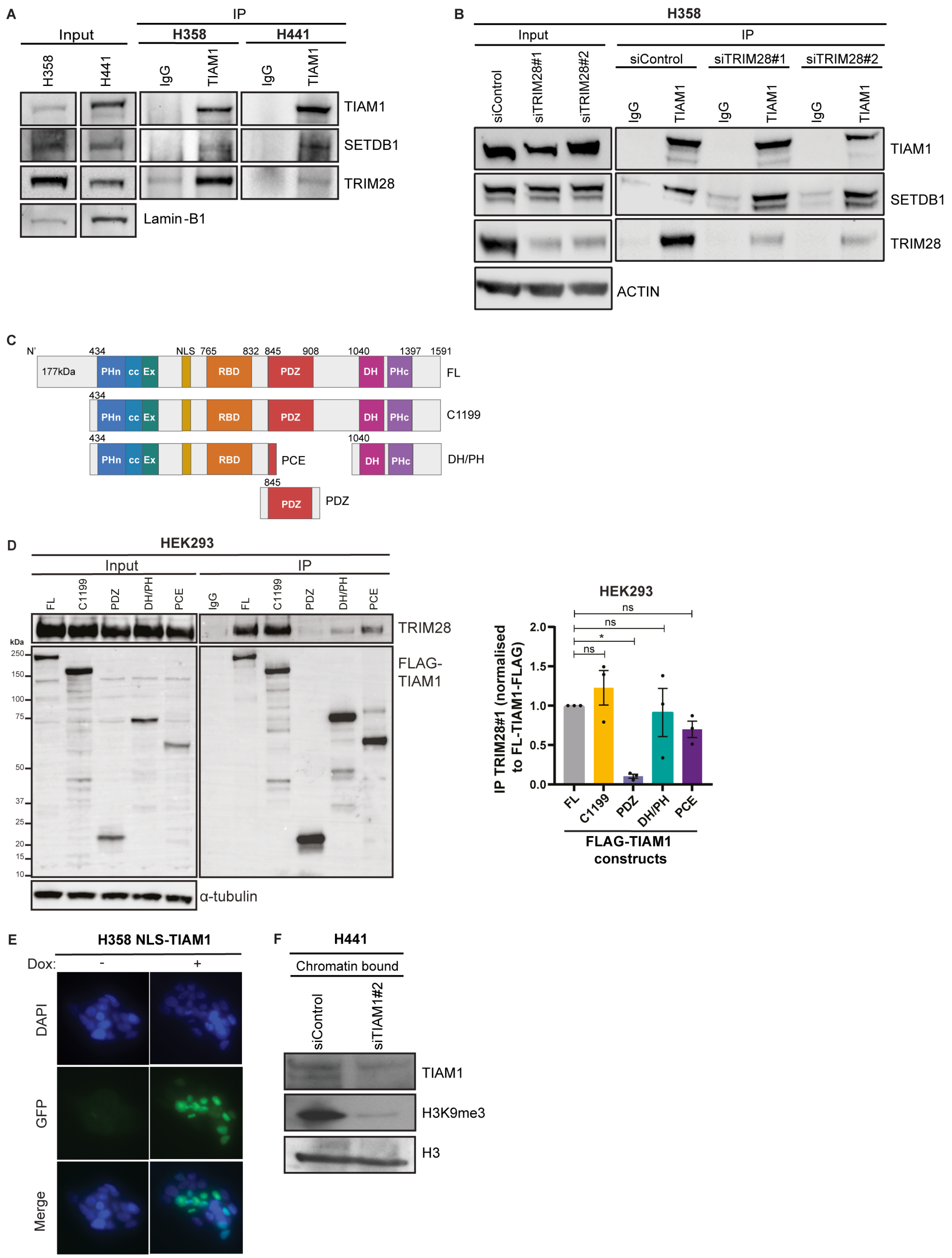

Fig. S5. TIAM1 interacts with TRIM28 and SETDB1

(A) Western blot analysis of endogenous TIAM1 co-immunoprecipitating with endogenous TRIM28 and SETDB1 from the nuclear fraction of H358 and H441 cells. (B) Western blot analysis of endogenous SETDB1 co-immunoprecipitating with endogenous TIAM1 in cells transfected with either control or TRIM28 siRNAs. (C) Schematic representation of FL-TIAM1 and TIAM1 deletion mutant constructs. (D) Western blot analysis of endogenous TRIM28 immunoprecipitated with exogenous flag-tagged TIAM1 deletion mutants in HEK293 cells. Graph quantifies TRIM28 immunoprecipitated with each TIAM1 deletion mutant normalised to TRIM28

immunoprecipitated with full-length TIAM1 (FL-TIAM1). (E) Fluorescence images of H358 cells stained with DAPI (blue) or showing epifluorescence of NLS-TIAM1 (GFP) and a merged overlay. Scale bar, 20 μm. (F) Western blot analysis of TIAM1 and H3K9me3 levels in the chromatin fraction of H441 cells transfected with TIAM1 or control siRNAs. H3 was used as a loading control. Data presented as mean ± SEM of three independent experiments. ns = non-significant, * $p<0.05$ (ANOVA).

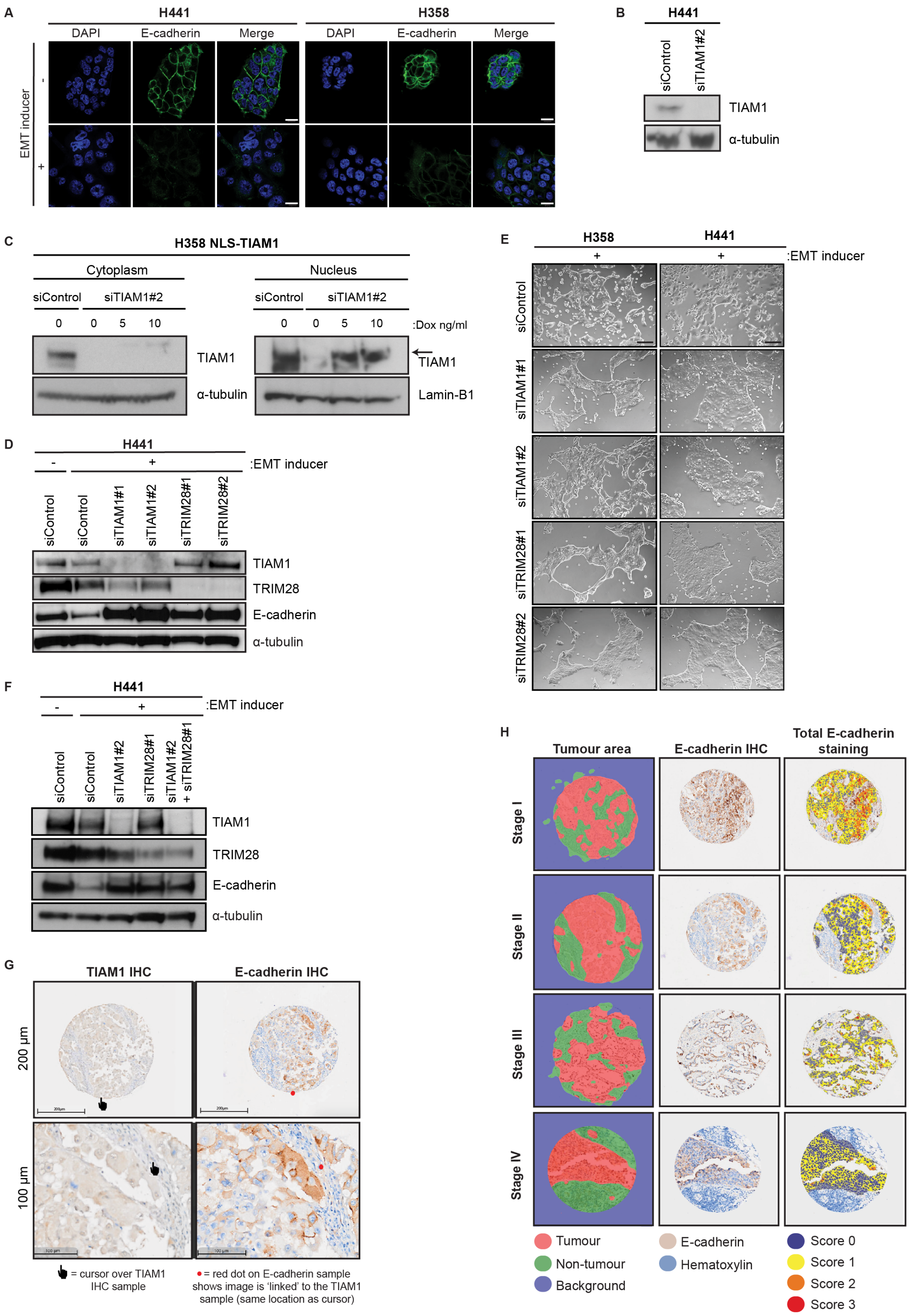

Fig. S6. TIAM1 regulates cell-cell adhesion

(A) Immunofluorescence images of H441 and H358 cells stained with DAPI (blue) and for E-cadherin (green) with a merged overlay in the presence (+) or absence (-) of EMT inducer. Scale bar, 20 μm. (B) Western blot analysis of TIAM1 levels in H441 cells transfected with either TIAM1 or control siRNAs used for the RNA seq experiment. (C) Western blot analysis of TIAM1 protein levels in cytoplasmic and nuclear fractions of H358 cells after transfection with TIAM1 or control siRNAs and induction of NLS-TIAM1 expression with doxycycline. (D) Western blot analysis of E-cadherin protein levels in H441 cells after treatment with EMT inducer (+) and transfection with

control, TIAM1 or TRIM28 siRNAs. (E) Bright-field images of H358 and H441 cells treated with control, TIAM1 or TRIM28 siRNAs and EMT inducer. Scale bar, 200 μm. (F) Western blot analysis of E-cadherin protein levels in H441 cells after treatment with EMT inducer (+) and transfection with control, TIAM1 and/or TRIM28 siRNAs. (G) Representative images showing image linking for TIAM1 and E-cadherin IHC serial sections of patient samples. Scale bar, 100 or 200 μm. (H) Representative images from the HALO AI E-cadherin IHC analysis pipeline. LUAD patient samples stained for TIAM1 were used to classify tumour areas from non-tumour areas. Tumour areas were matched to E-cadherin-stained samples (as in G) and analysers were then generated to detect total E-cadherin staining intensity within tumour areas (scores 0-3).

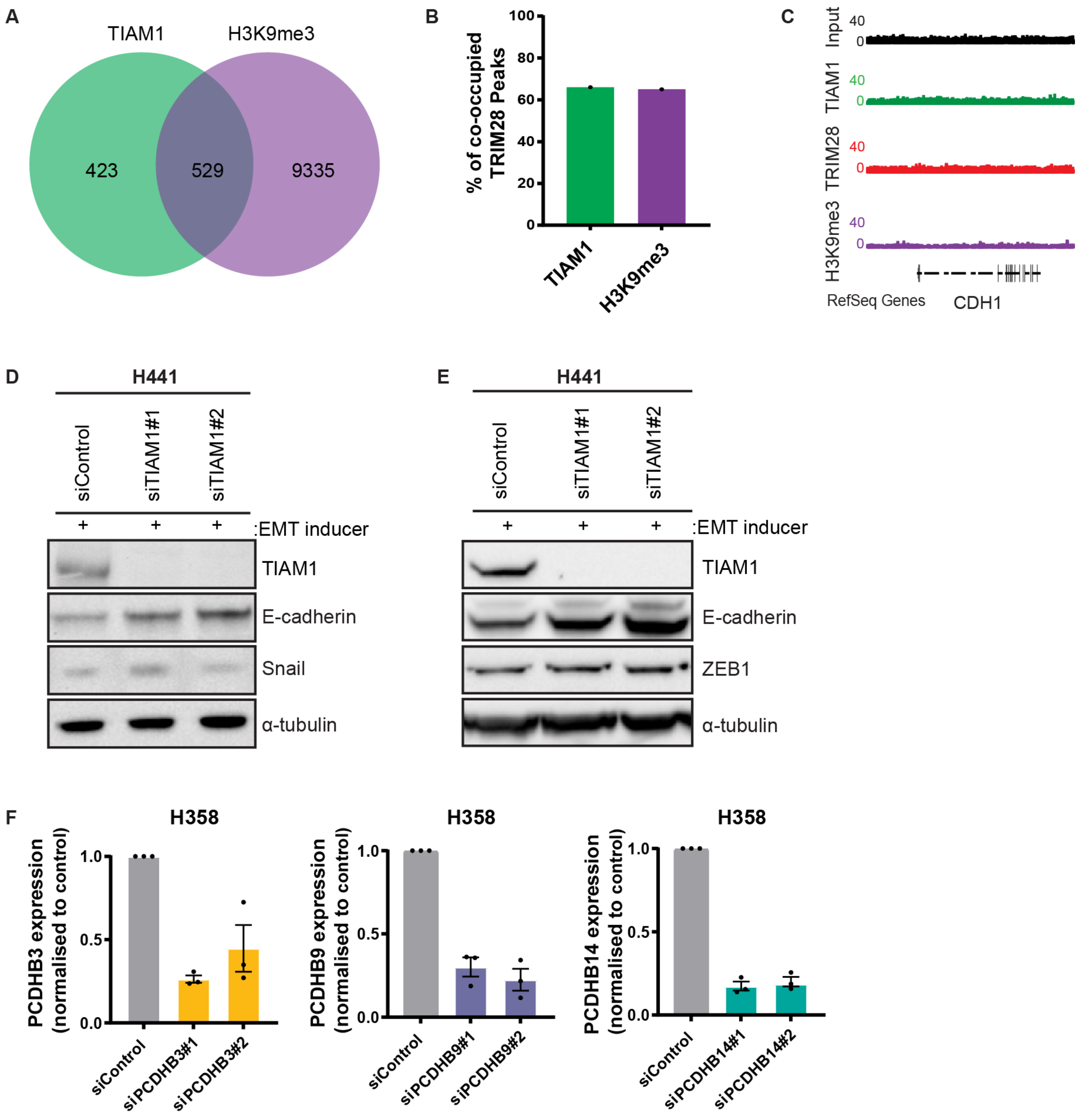

Fig. S7. TIAM1 directly regulates protocadherin expression and indirectly regulates E-cadherin expression

(A) Venn diagram showing the number of TIAM1, H3K9me3 and shared binding peaks identified by ChIP-sequencing. (B) Quantification of the percentage (%) of TRIM28 binding peaks identified by ChIP-sequencing that are co-occupied by TIAM1 or H3K9me3. (C) Read density tracks of TIAM1, TRIM28 and H3K9me3 ChIP-sequencing at the CDH1 gene of H441 cells, input sample shown as a control. (D) Western blot analysis of E-cadherin and Snail levels in H441 cells after treatment with EMT inducer (+) and transfection with control or TIAM1 siRNAs. (E) Western blot analysis of E-cadherin and ZEB1 levels in H441 cells after treatment with EMT inducer (+) and transfection with control or TIAM1 siRNAs. (F) qRT-PCR analysis of knock-down efficiency of PCDHB3, PCDHB9 and PCDHB14 in H358 cells treated with different siRNAs.

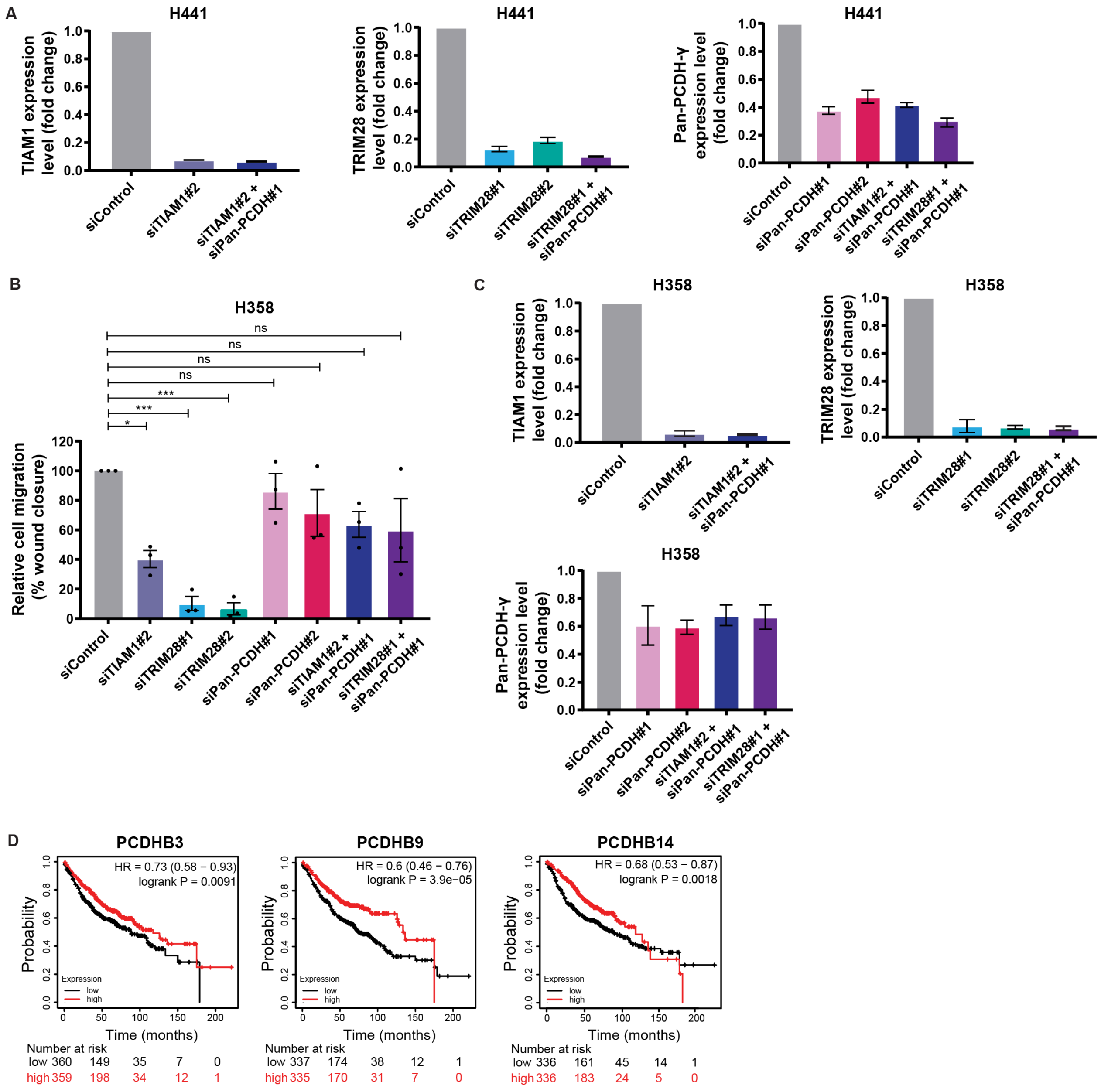

Fig. S8. TIAM1 promotes cell migration through protocadherins

(A) qRT-PCR analysis of knock-down efficiency of TIAM1, TRIM28 or pan-PCDH-γ in H441 cells treated with different siRNAs. (B) Graph shows percentage wound closure relative to initial wound size for H358 cells following transfection with control, TIAM1, TRIM28, pan-PCDHβ/γ siRNAs alone or combination. (C) qRT-PCR analysis of TIAM1, TRIM28 and pan-PCDH-γ expression levels in H358 cells. Graphs show expression levels normalised to control. (D) Kaplan–Meier curves generated from Kaplan-Meier plotter (kmplot.com) of overall survival of LUAD patients split by median PCDHB3, B9 or B14 expression. Hazard ratio (HR) and P values of the log-rank test are shown. Data presented as mean ± SEM of three independent experiments. ns = non-significant, * p<0.05, *** p<0.001 (ANOVA).

Supplementary Table 1

List of TIAM1 differentially expressed genes. (Provided as an Excel file)

Supplementary Table 2

Constructs used to generate stable cell lines.

**Construct label**	**Vector backbone**
Tet-On ®	pRetroXtight-Pur
TIAM1-HA	pRetroXtight-Pur
NLS-TIAM1-HA	pRetroXtight-Pur
BirA*-HA	pRetroXtight-Pur
NLS-BirA*-HA	pRetroXtight-Pur
TIAM1-BirA*-HA	pRetroXtight-Pur
NLS-TIAM1-BirA*-HA	pRetroXtight-Pur
NLS-TIAM1	pRetroXtight-Pur
NES-TIAM1	pRetroXtight-Pur
NLS-GD-TIAM1	pRetroXtight-Pur
TIAM1-FL	pFLAG-CMV2
TIAM1-DH-PH	pFLAG-CMV2
TIAM1-PDZ	pFLAG-CMV2
TIAM1-C1199	pFLAG-CMV2
TIAM1-PHn-CC-Ex	pFLAG-CMV2
shControl	LT3GEPIR
shTIAM1#1	LT3GEPIR
shTIAM1#2	LT3GEPIR

Supplementary Table 3

List of oligonucleotides used in this study.

Primer name	Sequence
β-Actin-FWD	5'-ATT GGC AAT GAG CGG TTC-3'
β-Actin-REV	5'-GGA TGC CAC AGG ACT CCA T-3'
GAPDH-FWD	5'-CCTGCACCACCAACTGCTTA-3'
GAPDH-REV	5'-CCATCACGCCACAGTTTCC-3'
TIAM1-FWD	5'-CCA TGA GCA GGG CAG TGT-3'
TIAM1-REV	5'-CGG AGA CGG CAT CAG AAT-3'
TRIM28-FWD	5'-GCG GAA AUG UGA GCG UGU A-3'
TRIM28-REV	5'-GCG AUC UGG UUA UGU GCA A-3'
E-cadherin-FWD	5'-CGG GGT CTC CCT GTG TTA C-3'
E-cadherin-REV	5'-TAC TCT GGG AGG CCA AGA TG-3'
pan-PCDH-γ-FWD	5'-CTG GCG TTT CTC TCA GGC CC-3'
pan-PCDH-γ-REV	5'-CAT GGC TTG CAG CAT CTC TG-3'
PCDHB3-FWD	5'-GGG TCC GAG TCA AGA CGC TAT-3'
PCDHB3-REV	5'-ATT ACT GTT CCT GGC AGA GTG C-3'
PCDHB9-FWD	5'-CAG GCT CCA GAA AAC AGT CC-3'
PCDHB9-REV	5'-TCT GCA TCT CCT GCA GAC AC-3'
PCDHB14-FWD	5'-TAG ACC GAG ACG AGC TGT GT-3'
PCDHB14-REV	5'-CTG TTG CTT CCG ACA TCC AAA-3'
**siRNA name**	**Sequence**
siControl	5'-CGU ACG CGG AAU ACU UCG A-3'
siTIAM1#1	5'-GAG ACU CCU CCG UAC AGU AAU UAU A-3'
siTIAM1#2	5'-CAG CAC AAC CCU GAC UGC GAC AUU-3'
siTRIM28#1	5'-GCG GAA AUG UGA GCG UGU A-3'
siTRIM28#2	5'-GCG AUC UGG UUA UGU GCA A-3'
sipan-PCDH-β/γ#1	5'-ACC UGG UGA CCA AGG UGG UGG CGG U-3'
sipan-PCDH-β/γ#2	5'-GAC UCG GGC CAG AAC GCC UGG CUG U-3'
siPCDHB3#1	5'-CGA GCU CCG UAU CAU AGA U-3'
siPCDHB3#2	5'-GGA CAG CCC AGA UAA ACA U-3'
siPCDHB9#1	5'-CCACUAUACGCAUUGUGGUCUUGGA-3'
siPCDHB9#2	5'-GAG AGC AAA GCU GAG UAC AAC UAC A-3'
siPCDHB14#1	5'-CGA UUA CAA AGA GAA UUC CAG AGA A-3'
siPCDHB14#2	5'-CCG GGA CAA AUG AGU UCA AAU UUC U-3'
**shRNA name**	**Guide sequence**
shControl	5'-TAG ATA AGC ATT ATA ATT CCT A-3'
shTIAM1#1	5'-TTG ACA AGC ATT TAC AAC GTT C-3'
shTIAM1#2	5'-TTT CTT AAA TTG ATC AAC CTT C-3'

Supplementary Table 4

List of antibodies used in this study and dilutions for each assay.

Antibody	Source	Identifier	Dilution/
Sheep anti-TIAM1	R&D Systems	AF5038	(WB 1:1000, IF 1:100, IHC 1:700, IP: 2-4μg/ml, ChIP: 1.6 μg)
Rabbit anti-TIAM1	Bethyl Laboratories	A300-009A	(WB 1:500)
Rabbit anti-TRIM28	Abcam	ab10484	(WB 1:1000, IF 1:100, IP: 4μg/ml, ChIP: 1.6 μg)
Rabbit anti-E-cadherin	Cell Marque	EP700Y (246R-14)	(IHC 1:200)
Rabbit anti-E-cadherin	Cell Signaling	24E10 (#3195)	(WB 1:1000, IF 1:100)
Rabbit anti-H3K9me3	Cell Signaling	D4W1U (#139695)	(WB 1:500, ChIP: 1.6 μg)
Rabbit anti-Histone 3	Cell Signaling	#9715	(WB 1:1000)
Rabbit anti-Lamin B1	Cell Signaling	D4Q4Z (#12586)	(WB 1:1000)
Mouse anti-GFP	Cell Signaling	#2955	(WB 1:1000)
Mouse anti-SETDB1	Abcam	ab107225	(WB 1:500)
Mouse anti-Lamin A/C	Abcam	ab8984	(WB 1:1000)
Mouse anti-α-tubulin	Sigma	T6199	(WB 1:1000)
Mouse anti-β-actin	Sigma	A1978	(WB 1:1000)
Mouse anti-FLAG	Sigma	F1804	(IP: 2μg/ml
Rabbit anti-FLAG	Sigma	F7425	(WB 1:2000)
Rabbit anti-ZEB1	Cell Signaling	D80D3 (#3396)	(WB 1:1000)
Rabbit anti-Snail	Cell Signaling	C15D3 (#3879)	(WB 1:1000)
Streptavidin HRP	Thermo	21130	(WB 1:5000)
Streptavidin-594	Invitrogen	S11227	(IF 1:500)
Rat anti-HA	Roche	11867423001	(WB 1:1000, IF 1:100)
Sheep HRP-anti Mouse	GE Healthcare	NA931	(WB 1:5000)
Donkey HRP-anti Sheep	Bio-Rad	STAR88P	(WB 1:5000)
Donkey HRP-anti Rabbit	GE Healthcare	NA934	(WB 1:5000)
Donkey 488-anti Sheep	Invitrogen	A11015	(IF: 1:500)
Goat 488-anti Rat	Invitrogen	A21208	(IF: 1:500)
Goat 488-anti Rabbit	Invitrogen	A11034	(IF: 1:500)
Goat 568-anti Rabbit	Invitrogen	A21428	(IF: 1:500)

References

1. S. Peters *et al.*, Lungscape: resected non-small-cell lung cancer outcome by clinical and pathological parameters. *J Thorac Oncol* **9**, 1675-1684 (2014).
2. R. Katayama *et al.*, Modulation of Wnt signaling by the nuclear localization of cellular FLIP-L. *J Cell Sci* **123**, 23-28 (2010).
3. S. A. Woodcock *et al.*, SRC-induced disassembly of adherens junctions requires localized phosphorylation and degradation of the rac activator tiam1. *Mol Cell* **33**, 639-653 (2009).
4. C. Fellmann *et al.*, An optimized microRNA backbone for effective single-copy RNAi. *Cell Rep* **5**, 1704-1713 (2013).
5. A. Lánczky, B. Győrffy, Web-Based Survival Analysis Tool Tailored for Medical Research (KMplot): Development and Implementation. *J Med Internet Res* **23**, e27633 (2021).
